# A Ran-binding protein facilitates nuclear import of human papillomavirus type 16

**DOI:** 10.1371/journal.ppat.1009580

**Published:** 2021-05-11

**Authors:** Kun-Yi Lai, Matteo Rizzato, Inci Aydin, Ruth Villalonga-Planells, Hannes C. A. Drexler, Mario Schelhaas

**Affiliations:** 1 Institute of Cellular Virology, Westphalian Wilhelms-University of Münster, Münster, Germany; 2 Interfaculty Centre ‘Cells in Motion’ (CiM), Westphalian Wilhelms-University of Münster, Münster, Germany; 3 Biomolecular Mass Spectrometry Unit, Max Planck Institute for Molecular Biomedicine, Münster, Germany; Penn State University School of Medicine, UNITED STATES

## Abstract

Human papillomaviruses (HPVs) utilize an atypical mode of nuclear import during cell entry. Residing in the Golgi apparatus until mitosis onset, a subviral complex composed of the minor capsid protein L2 and viral DNA (L2/vDNA) is imported into the nucleus after nuclear envelope breakdown by associating with mitotic chromatin. In this complex, L2 plays a crucial role in the interactions with cellular factors that enable delivery and ultimately tethering of the viral genome to mitotic chromatin. To date, the cellular proteins facilitating these steps remain unknown. Here, we addressed which cellular proteins may be required for this process. Using label-free mass spectrometry, biochemical assays, microscopy, and functional virological assays, we discovered that L2 engages a hitherto unknown protein complex of Ran-binding protein 10 (RanBP10), karyopherin alpha2 (KPNA2), and dynein light chain DYNLT3 to facilitate transport towards mitotic chromatin. Thus, our study not only identifies novel cellular interactors and mechanism that facilitate a poorly understood step in HPV entry, but also a novel cellular transport complex.

## Author summary

Human papillomaviruses (HPVs) cause proliferative lesions such as benign warts or malignant invasive cancers. Like other DNA viruses, HPV has to deliver its genome to the nucleus for viral genome transcription and replication. After initial attachment, HPVs are endocytosed to be eventually directed to the trans-Golgi-network (TGN) by intracellular trafficking, where they reside until cell division. Mitosis onset enables access of the virus to cellular chromatin after nuclear envelope breakdown. Tethering of the virus to mitotic chromatin ensures nuclear delivery upon reformation of the nuclear envelope after mitosis. Our previous work showed that the minor capsid protein L2 facilitates nuclear delivery. However, the detailed mechanism, namely, how HPV trafficks from cytosol to the nuclear space, is barely understood. Here, we identified for the first time cellular proteins that interacted with L2 for nuclear import. Mechanistically, the proteins formed a hitherto unknown cellular transport complex that interacted with L2 to direct the virus to mitotic chromosomes by microtubular transport. Our findings provided not only evidence for a transport mechanism of a poorly understood step of HPV entry, but also discovered a novel cellular transport complex.

## Introduction

Human papillomaviruses (HPVs) are a large family of small non-enveloped DNA viruses. Their icosahedral (T = 7) capsids are composed of 72 pentameric capsomers of the major capsid protein L1 and up to 72 molecules of the minor capsid protein L2 [1–4]. The capsid encloses an 8 kb double-stranded, chromatinized DNA genome [5]. HPVs type-dependently infect skin or mucosal epithelia [6–8]. Infection of HPVs may cause proliferative lesions such as benign warts, or malignant invasive cancers. HPV type 16 and 18 are the etiological agents of cervical cancers [9–11]. Together with other types, HPV16 and HPV18 are considered as high-risk types for the development of anogenital cancers, and are amongst the primary targets for vaccination [12].

As most DNA viruses, HPV16 needs to deliver its genome to the host nucleus during cell entry to allow viral gene expression and genome replication, which typically comprises multiple steps from receptor binding to nuclear import. Most steps during the HPV16 entry program have been identified and initially characterized over the last years. However, many of those steps remain only partially understood. For HPV16, entry starts with binding to heparan sulphate proteoglycans (HSPGs) within the plasma membrane or the extracellular matrix (ECM) [13–17]. This binding facilitates a sequence of subsequent conformational and proteolytic changes that activate the virus for infectious uptake [18]. Activation exposes previously masked epitopes of L1 and L2, which are in turn cleaved by the extracellular proteases kallikrein-8 and furin, respectively [19, 20]. The cumulative changes are hypothesized to facilitate interaction with an elusive secondary receptor or receptor complex [21]. Engagement is thought to trigger endocytosis through growth factor receptor and Abl2 signalling from tetraspanin-enriched microdomains by a novel actin-dependent pathway [21–24]. After endocytosis, HPV16 is routed to late endosomal compartments, where the major capsid protein L1 at least partially separates from a subviral complex composed of the minor capsid protein L2 and viral DNA (L2/vDNA) [25–27]. While the extent of how much L1 remains associated with L2/vDNA is still under debate [28, 29], it is clear that L2 interactions are crucial for further steps during entry. In endosomes, L2 is able to penetrate the limiting membrane into the cytosol with its C-terminal domain [30]. This facilitates the interactions with cellular trafficking complexes such as the retromer, sorting nexins, and γ-secretase which address L2/vDNA to the trans-Golgi network (TGN) [31–36].

Nuclear import of HPV16, namely trafficking of L2/vDNA from the TGN to the intranuclear space, occurs upon nuclear envelope breakdown (NEBD) during mitosis [37, 38]. Throughout this phase, it has been suggested that L2/vDNA remains enclosed within Golgi-derived vesicles [39]. Upon vesiculation, it appears that additional L2 C-termini penetrate the limiting membrane [40]. These vesicles containing the subviral complex eventually tether to condensed mitotic chromosomes [40, 41]. While we established that a central peptide of L2 termed the chromosomal binding region (CBR) is crucial to tether the complex to mitotic chromatin [41], it is still unclear which cellular factors or mechanisms facilitate the second membrane penetration event, and direct the subviral complex to mitotic chromatin. However, since L2/vDNA is found associated with mitotic microtubules (MTs) and the microtubule-organizing center (MTOC) [40, 42], and since MT-dependent motor proteins, such as dynein light chains (DYNLT1 and DYNLT3), can interact with HPV16 L2 [43, 44], a MT-dependent transport towards mitotic chromatin is plausible.

In this study, we aimed to determine host factors involved in nuclear import during mitosis. For this, a label-free mass spectrometry approach was used to identify potential cellular L2 interaction partners during mitosis. Using virological, biochemical and microscopy approaches in combination with RNAi silencing or pharmacological inhibition, we determined that one of the candidates, namely Ran-binding protein 10 (RanBP10) in a complex with karyopherin alpha 2 (KPNA2) and dynein light chain DYNLT3 facilitated nuclear delivery of L2/vDNA most likely through microtubular transport during mitosis.

## Results

### A label-free mass spectrometry approach identifies RanBP10 as a potential interactor of L2 during mitosis

**Fig 1:**
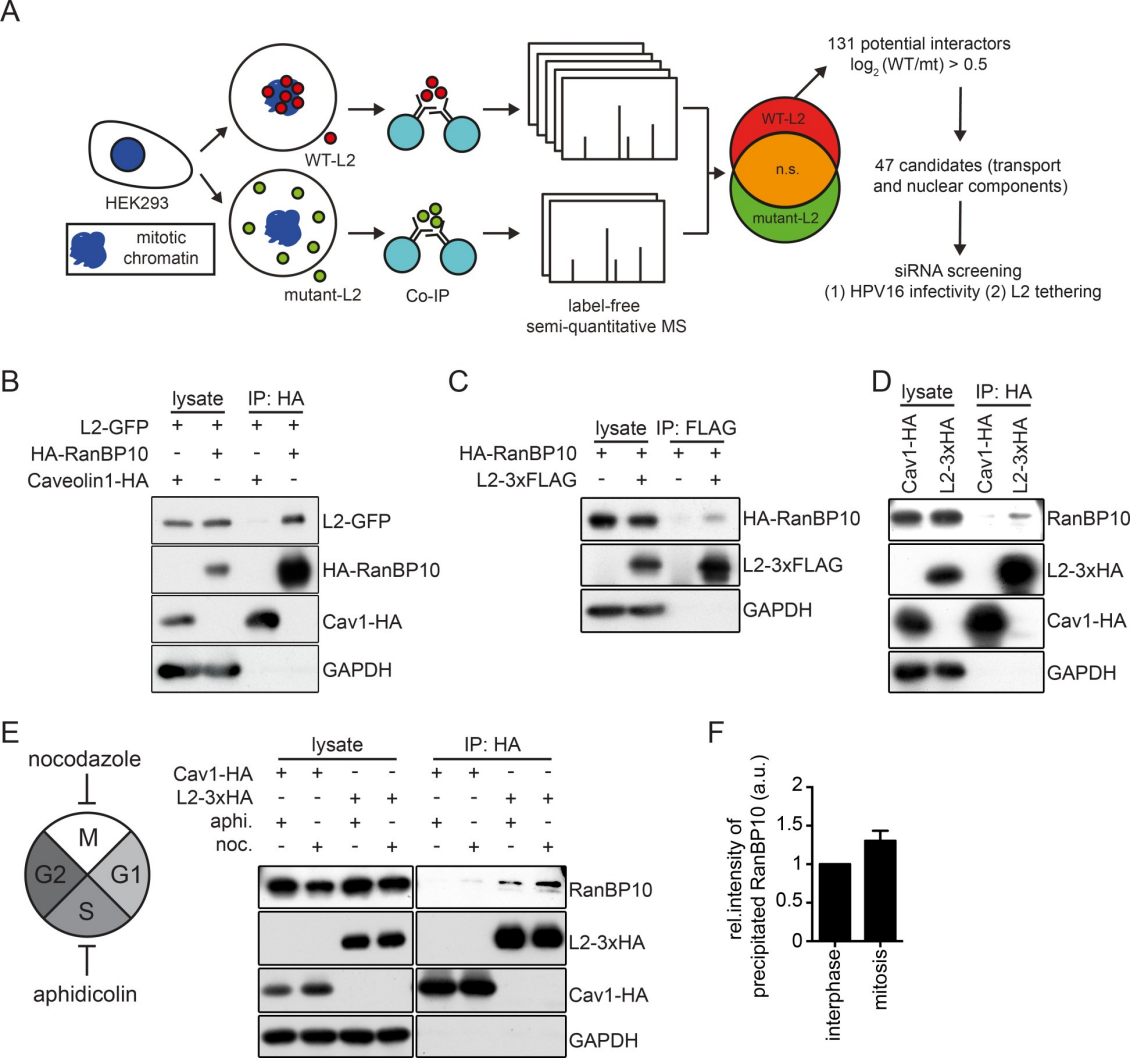
Identification of a novel L2 interaction partner during mitosis. **(A)** Overview of the experimental strategy to identify mitotic L2 interactors using label-free semi-quantitative mass spectrometry. HEK293 cell lysates expressing wild-type L2-3xHA or RTR313EEE-mutant L2-3xHA were subjected to immunoprecipitation with an HA antibody. Cellular proteins co-precipitating with L2 were identified by label-free semi-quantitative mass spectrometry and analyzed with MaxQuant v1.5.3.12. 47 candidates were selected and preliminarily tested on their functional roles in HPV16 infectivity and L2 chromatin association. **(B)** Immunoprecipitation of HEK293 cell lysates co-expressing L2-GFP and HA-RanBP10. Caveolin 1-HA (Cav1-HA) was used as a negative control for the HA-tag pull-down. **(C)** Reverse pull-down for (B). Immunoprecipitation of HEK293 cell lysates co-expressing RanBP10 and L2-3xFLAG with a FLAG-tag antibody. **(D)(E)** Immunoprecipitation against HA in (D) unsynchronized or (E) synchronized HEK293 cells expressing L2-3xHA, followed by detection of endogenous RanBP10. Cells were synchronized for 16 hours with either aphidicolin (3 μM) to result in interphase-arrested cells or nocodazole (330 nM) to result in prometaphase-arrested cells. Caveolin 1-HA (Cav1-HA) was used as a negative control. **(F)** Quantification of RanBP10 immunoprecipitates from **(E)** of three independent experiments. Given are values relative to interphase levels ± SD.

To date, it remains largely unclear, which cellular processes or cellular L2 interaction partners promote nuclear import of the vDNA during mitosis. To address this question we first investigated, which cellular proteins physically interact with L2 during mitosis. For this, we used a biochemical co-immunoprecipitation (co-IP)/mass-spectrometry assay with subsequent label-free quantification. In this approach, we quantitatively compared cellular proteins that co-immunoprecipitated with either wild-type (WT) L2 or a L2 mutant (RTR313EEE) incapable of associating with mitotic chromatin during mitosis [41]. Proteins that more abundantly co-immunoprecipitated with WT-L2 but not mutant L2 were considered as candidates facilitating nuclear import (Figs 1A and S1A and S1 Table, and material and methods). Under low stringency conditions, we identified 131 proteins that were enriched for interaction with WT L2 as compared to RTR313EEE L2 (log_2_>0.5). Of those, 47 candidates were selected for downstream analysis, since their functions were related to mitosis or nuclear processes (Fig 1A and S1 Table). Upon siRNA-mediated knockdown of the respective proteins, the contribution of candidates to infection and L2 association was tested in two separate assays. Knockdown of few candidates exhibited reduced infection and association with mitotic chromatin (S1 Table). Of those, we decided to analyze one particular candidate in depth, because it displayed the strongest phenotype.

This candidate protein, namely Ran-binding protein 10 (RanBP10) is understudied and little is known in terms of its cellular function. Ran-binding proteins comprise a diverse group of proteins with an equally diverse range of cellular functions. Classically, they facilitate nuclear import and export, but additional cytoplasmic roles have emerged over the past two decade including interactions with MTs [45]. RanBP10 has been dubbed a Ran-binding protein due to its sequence similarity with Ran-binding protein 9 (RanBP9), both of which comprise atypical functions for RanBPs. Some initial work points to RanBP10’s roles in maintaining cytosolic Ran activity as a guanine nucleotide exchange factor (GEF) in megakaryocytes to facilitate nuclear transport and in interacting with MTs during interphase [46–48]. RanBP10 is also found to act as a co-activator for androgen receptor accompanied by Ran-binding protein M (RanBPM) and as a part of the multi-subunit C-terminal to LisH complex [49]. From these initial reports, however, a clear functional role of RanBP10 does not emerge.

To characterize the biological functions of RanBP10 and its potential role(s) in HPV16 nuclear import, we first confirmed that RanBP10 interacted with L2. In HA-RanBP10- and L2-GFP-expressing HEK293 cells, L2 was able to precipitate RanBP10 confirming a physical interaction (Fig 1B and 1C). In addition, L2 also interacted with endogenous RanBP10 (Fig 1D). L2 interacted with RanBP10 in cells arrested during interphase and slightly more pronounced in cells arrested in mitosis (Fig 1E and 1F). Overall, these data confirmed the mass spectrometry data on the interaction of RanBP10 with L2 during mitosis.

To assess the subcellular localization of the RanBP10-L2 interaction, we microscopically analyzed the co-localization of overexpressed HA-RanBP10 with L2-GFP during different phases of the cell cycle. In HeLa cells, overexpressed L2 localized during interphase exclusively to the nucleus with dot-like accumulations that presumably represented PML nuclear bodies (S1C Fig) [50, 51]. Overexpressed HA-RanBP10 was mainly localized to the cytosol in interphase cells (S1C Fig, middle row). In addition to a homogenous distribution throughout the cytosol, RanBP10 notably displayed dot-like accumulations particularly within the perinuclear area. Upon co-expression, HA-RanBP10 and L2-GFP, we observed co-localizing spots of HA-RanBP10 and L2-GFP in the perinuclear area (S1C Fig). This result indicated that RanBP10 was able to associate with and re-localize L2-GFP. During mitosis, however, HA-RanBP10 was dispersed throughout the cytosol and did not associate with mitotic chromatin contrary to L2 (S1C Fig). This suggested that RanBP10 was not tethering the incoming vDNA to mitotic chromosomes during prometaphase. Hence, we hypothesized that RanBP10 interacts with cytosolic L2 during the transport of L2/vDNA to mitotic chromatin during mitosis.

### RanBP10 is important for HPV16 infection and L2 chromatin association

**Fig 2:**
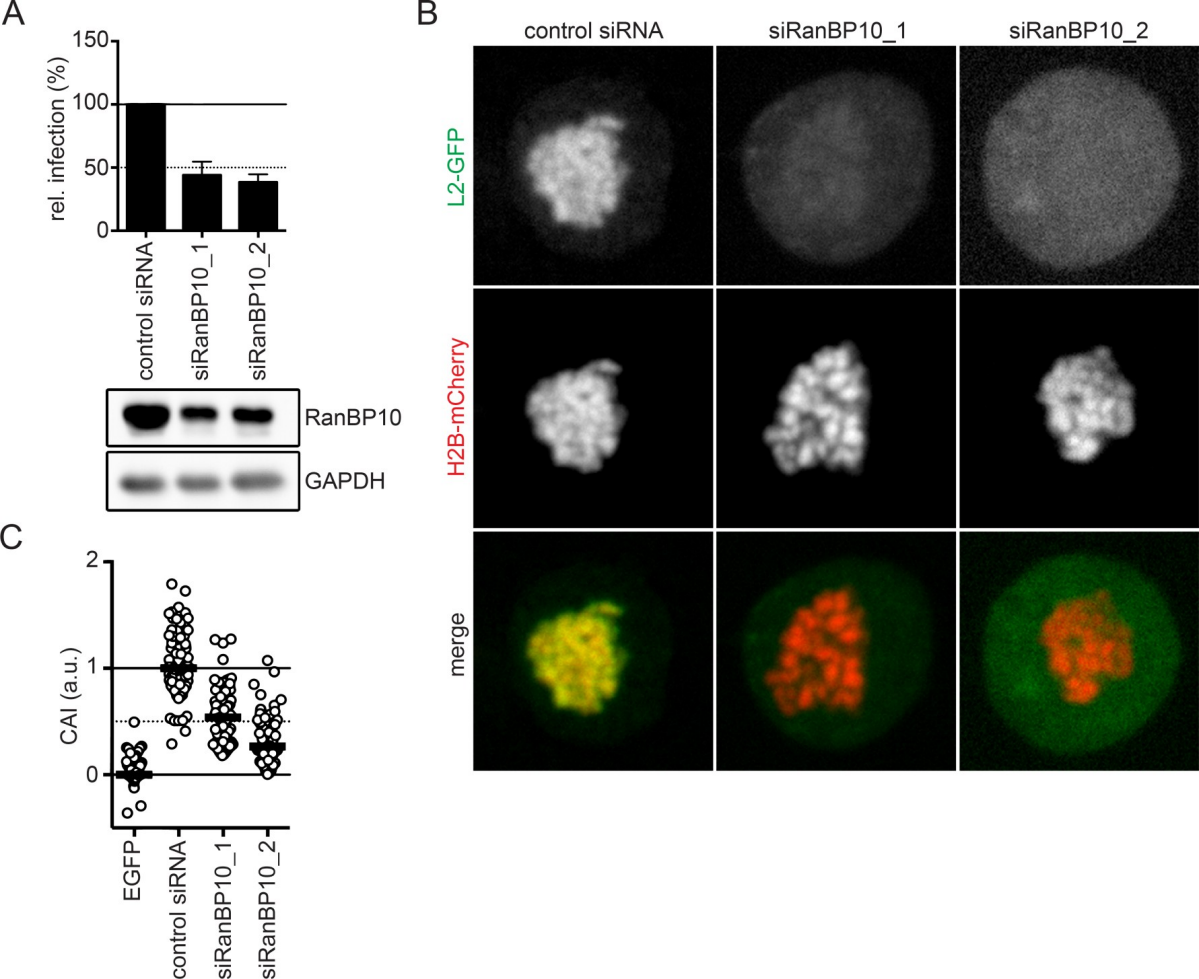
A crucial role of RanBP10 for HPV16 infectivity and L2 tethering. **(A)** RNAi of RanBP10 in HeLa cells was followed by HPV16-PsV infection for 48 hours. Infectivity was scored by flow cytometry based on the percentage of the cells expressing GFP. The infectivity was normalized to control siRNA transfected cells and depicted as relative (rel.) infection. The protein expression level of RanP10 upon siRNA knockdown was analyzed by Western blotting. **(B)** RNAi of RanBP10 in HeLa Kyoto_H2B-mCherry_L2-GFP cells was followed by arrest in prometaphase using nocodazole (330 nM) for 16 hours. Cells were fixed and images were acquired using a spinning disk confocal microscope. Depicted are single median slices. **(C)** Analysis of **(B)** quantifying the degree of chromosomal association as described in material and methods. Displayed is the chromosomal association index (CAI) relative to control siRNA-treated cells (1) and GFP expressing cells (0). In three independent experiments, at least 50 cells were analyzed. The median was indicated by a black bar.

To confirm that the interaction was critical for HPV16 entry, infectivity of HPV16 pseudoviruses (PsVs) was tested upon knockdown of RanBP10 in HeLa cells. HPV16 PsVs consist of HPV16 particles harbouring a GFP expressing pseudogenome [52, 53]. HPV16 infectivity is assessed by the number of GFP expressing cells. HPV16 infection was reduced to about 50% upon knockdown of RanBP10 using two independent siRNAs (Fig 2A). Since RNAi of RanBP10 reduced protein levels only to about 30% of the control (Fig 2A), we tested whether further decreasing RanBP10 levels would impair HPV16 infection further. Interestingly, HPV16 infectivity was not further impaired even upon almost complete depletion of RanBP10 (S1B Fig). While this established the importance of RanBP10 for HPV16 entry, it also suggested that its contribution was partial.

Next, we aimed to verify the importance for nuclear delivery. Nuclear delivery of the HPV16 subviral complex (L2/vDNA) is mediated by L2: incoming vDNA from HPV16 PsVs associates with mitotic chromosomes during prometaphase/metaphase, and ectopically expressed L2 is recruited to mitotic chromatin phenocopying the vDNA [38, 41]. Thus, L2 tethers the viral genome to mitotic chromosomes upon NEBD. To initially assess whether RanBP10 affected L2 association with mitotic chromosomes, we used the L2 chromosomal association assay, in which ectopically expressed L2 associates with mitotic chromatin, and quantified the amount of L2 that associated with mitotic chromatin upon depletion of RanBP10 in comparison to control cells. Similar to our infectivity data, L2 associated about 50% less with mitotic chromosomes upon knockdown of RanBP10 in comparison to the control (Fig 2B and 2C). Thus, the reduction in HPV16 infectivity was correlated with a decrease in L2 association with mitotic chromatin. Taken together, these data suggested that RanBP10 played an important role in HPV16 nuclear import.

### RanBP10 facilitates nuclear delivery of vDNA

**Fig 3:**
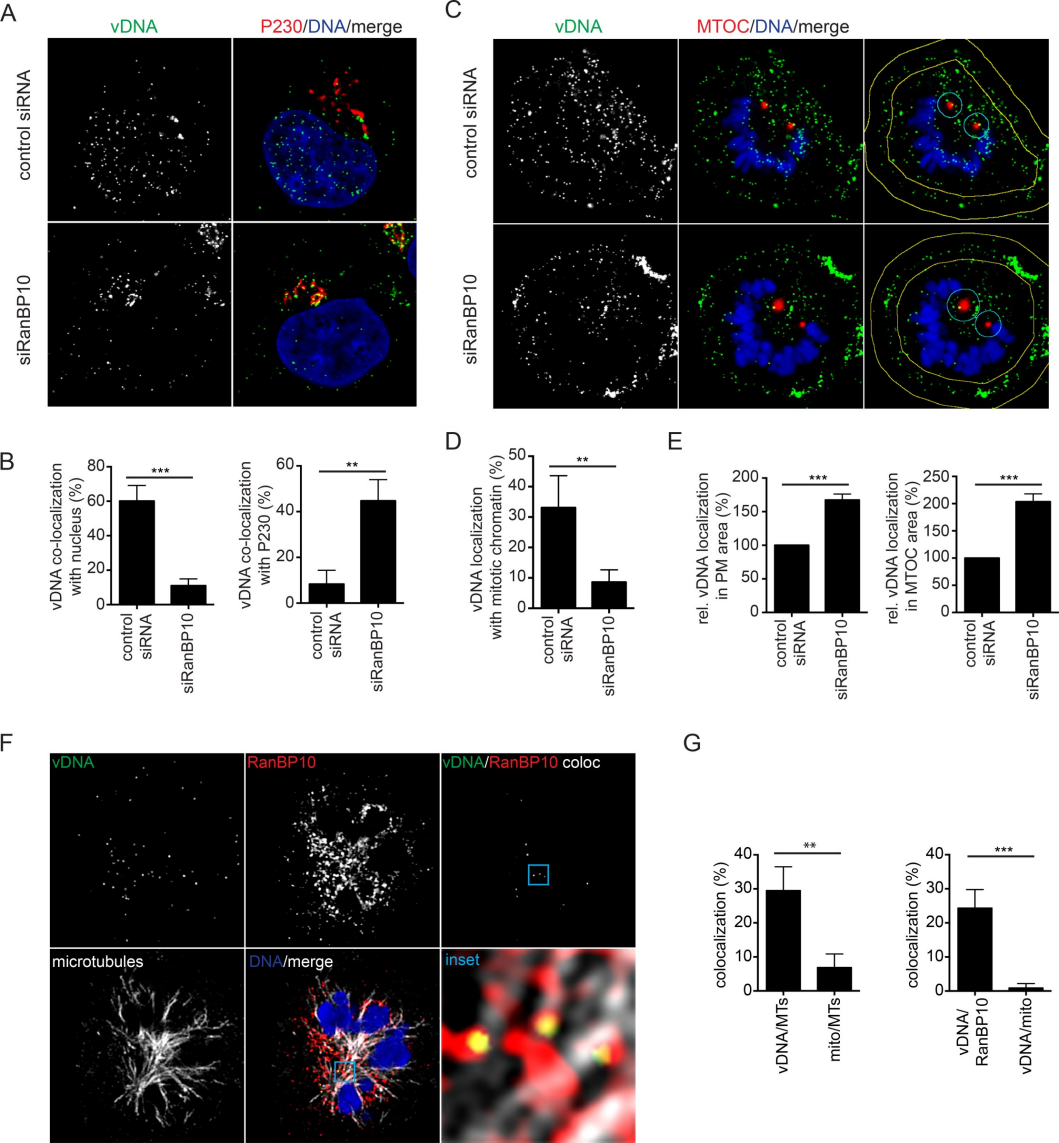
RanBP10 facilitated nuclear delivery of incoming HPV16 viral DNA. vDNA from incoming EdU-labelled HPV16 PsV was detected using EdU Click-iT chemistry, nuclei were stained by Hoechst-33258. All images were acquired by confocal microscopy. Images represent single medial slices. The degree of overlapping signals from at least three independent experiments was quantified using IMARIS. **(A)** RNAi of RanBP10 in HeLa cells was followed by infection with EdU-labelled HPV16. Cells were fixed at 20 h.p.i., and stained with an anti-P230 antibody and Hoechst-33258 to indicate the TGN and nucleus, respectively. **(B)** Quantification of co-localized vDNA signals with the nucleus or P230. At least 35 cells were analyzed in three independent experiments. The error bars indicate the SD. **(C)** Experiments as in **(A)** were performed in mitotically arrested cells. Yellow lines in the right column indicate margins for quantification of **(E)**. **(D)** Quantification of co-localized vDNA on mitotic chromosomes. At least 35 cells were analyzed in three independent experiments. The error bars indicate the SD. **(E)** Quantification of vDNA localized to plasma membrane or MTOC area normalized to control siRNA-treated cells. **(F)** Co-localization of endogenous RanBP10 with incoming vDNA and MTs. HeLa cells were infected with EdU-labelled HPV16 and arrested in mitosis. Cells were stained for vDNA, endogenous RanBP10 and alpha-tubulin (MTs). The channel indicating co-localized signals of vDNA and RanBP10 (top right) was generated using IMARIS. **(G)** Quantification of co-localized vDNA with either MTs (left) or RanBP10 (right). Indicated is also coincidence overlap with mitochondria (mito), images not shown. At least 35 cells were analyzed in three independent experiments.

To test whether RanBP10 was indeed involved in nuclear delivery of vDNA from incoming viruses, we used EdU-labelled HPV16 PsVs to infect RanBP10-depleted cells [41]. In control siRNA-transfected cells, about 60% and 8.5% of the incoming vDNA signals localized to the nucleus and Golgi at 20 h.p.i, respectively (Fig 3A and 3B). Notably, the distribution was reversed in RanBP10-depleted cells with 11% and 45% of vDNA localizing to the nucleus or Golgi, respectively (Fig 3A and 3B). This indicated that while nuclear import was facilitated by the presence of RanBP10, trafficking to the Golgi was unaffected in the absence of RanBP10.

Tethering of L2/vDNA to mitotic chromatin occurs during prometaphase/metaphase [38, 41]. To verify whether tethering of incoming vDNA to mitotic chromatin would require RanBP10 as indicated by our data on L2 chromatin association, the localization of vDNA was quantified in mitotic cells at 20 h.p.i. In control siRNA-treated cells, about 35% of the vDNA signal localized to condensed mitotic chromatin, whereas only 8% of vDNA showed overlap with mitotic chromatin in RanBP10-depleted cells (Fig 3C and 3D). Instead of localizing to mitotic chromatin, the vDNA signals exhibited a different intracellular distribution in RanBP10-depleted cells: they were less dispersed but localized more closely to regions proximal to the plasma membrane and the MTOC (Fig 3C and 3E) suggesting a transport-related perturbation. In summary, the data indicated that RanBP10 was involved in delivering vDNA from the Golgi to mitotic chromatin.

To evaluate whether RanBP10 played a direct role in the delivery of vDNA from the Golgi to the nucleus, we further asked whether endogenous RanBP10 would associate with incoming vDNA during prometaphase. In cells synchronized for progression into mitosis, about 25% of incoming vDNA co-localized with endogenous RanBP10 during prometaphase as assessed by confocal microscopy of EdU-labelled HPV16 (Fig 3F and 3G). Notably, co-localized vDNA/RanPB10 did not overlap significantly with mitotic chromatin or the plasma membrane. Thus, these findings suggested that RanBP10 itself did not tether L2 to chromatin in line with RanBP10’s absence on mitotic chromatin in L2 expressing cells (S1C Fig), nor that it played a role in initial uptake of the virus in line with successful vDNA delivery to the Golgi apparatus (Fig 3E and 3F). Instead, the overlapping signals were significantly localized to mitotic MTs when assessed by confocal or super-resolution radial fluctuations (SRRF) microcopy and compared to coincidental overlap with mitochondria (Fig 3F, 3G, S2A and S2B). This suggested that RanBP10 facilitated trafficking of L2/vDNA along MTs towards mitotic chromatin.

### RanBP10 mediates L2 interaction with KPNA2

To shed some light on how RanBP10 may facilitate L2/vDNA transport along MTs, we focused our attention on MT-dependent motors and their adaptor proteins. MT-dependent motor proteins function as transporters not only during interphase but also during mitosis (reviewed in [54, 55]). Importin subunits have been shown to interact with these transporters along with cellular adaptor proteins, such as Targeting Protein for Xklp2 (TPX2), to deliver cellular cargo along MTs and eventually reach mitotic chromatin [56–58]. Previous work indicated that L2 co-immunoprecipitates with karyopherin 2 (KPNA2) [59]. Moreover, dynein motors, more specifically dynein light chains, associate with incoming HPV16 L2 during infection [43]. While both findings have not been related to mitosis, these reports together with the localization of incoming L2/vDNA complex to mitotic MTs [39] led us to hypothesize that L2/vDNA may traffic with the help of a RanBP10/KPNA2/motor protein complex during mitosis.

**Fig 4:**
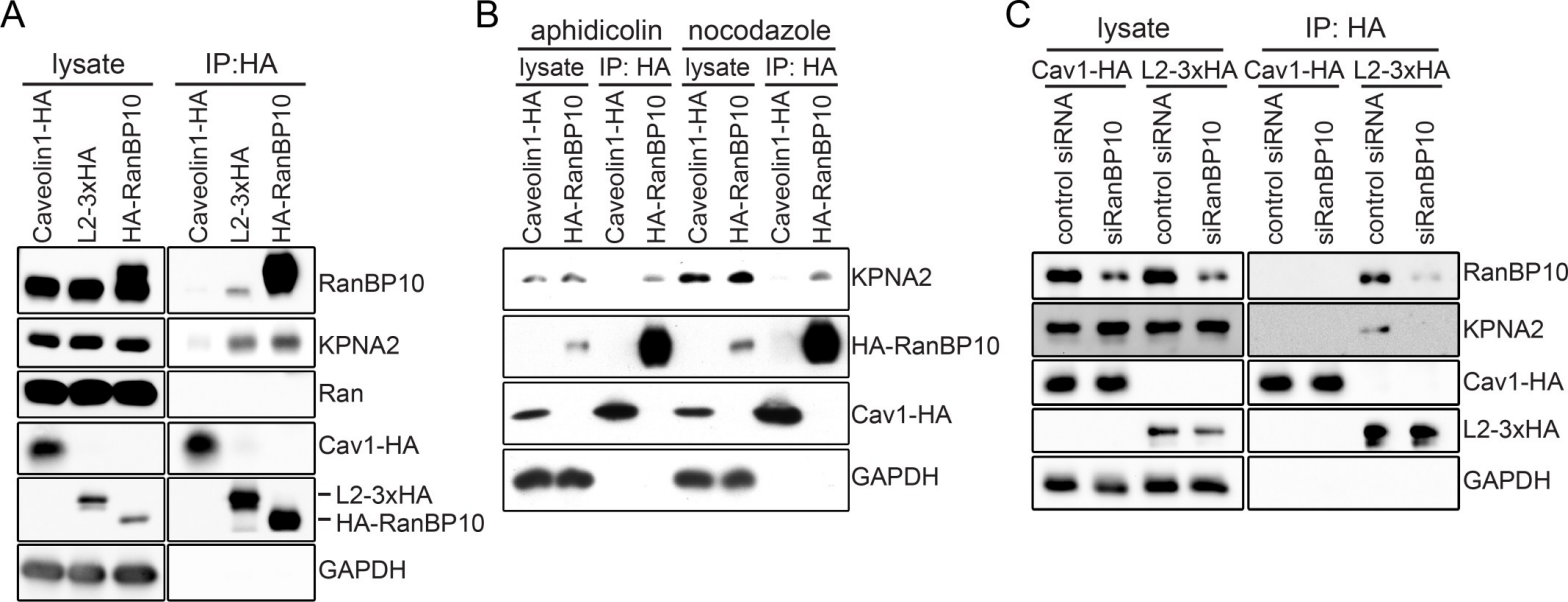
L2 and RanBP10 formed a complex with KPNA2. Immunoprecipitation was used to determine potential interactions between L2, RanBP10 and KPNA2 in HEK293 and HeLa cells. **(A)** Caveolin1-HA, L2-3xHA or HA-RanBP10 were ectopically expressed in HEK293 cells, and immunoprecipitation against HA was performed on cell lysates. Caveolin1-HA was used as a negative control of immunoprecipitation. Endogenous RanBP10, KPNA2, and Ran were detected by Western blotting after immunoprecipitation. **(B)** aphidicolin (3 μM) or nocodazole (330 nM) were used to arrest HEK293 cells ectopically expressing caveolin1-HA, or HA-RanBP10 in interphase or mitosis, respectively. Cell lysates were subjected to immunoprecipitation against HA and endogenous KPNA2 was detected by Western blotting. **(C)** HeLa cells were transfected with RanBP10 siRNA, followed by ectopic L2-3xHA expression for 24 hours. Cell lysates were subjected to immunoprecipitation against HA. Caveolin1-HA was used as a negative control for HA-tag pull-down. Endogenous RanBP10 or KPNA2 were detected by Western blotting.

To test this hypothesis, we first investigated whether RanBP10 would interact with KPNA2. In L2-3xHA or HA-RanBP10 overexpressing cells, endogenous KPNA2 precipitated with both proteins in interphase cells (Fig 4A). Furthermore, RanBP10 pulled down KPNA2 also during mitosis (Fig 4B). This data suggested that L2 interacted with a complex composed of RanBP10 and KPNA2 (RanBP10/KPNA2). While signals of endogenous RanBP10 and KPNA2 showed overlap in confocal micrographs (S3A Fig), the high and disperse cytosolic abundance of KPNA2 during mitosis renders quantification uninformative as it cannot be distinguished from coincidental overlap. To further dissect the interaction between L2 and RanBP10/KPNA2, endogenous RanBP10 was depleted followed by overexpression of L2-3xHA. L2 interacted less with endogenous KPNA2, if RanBP10 was depleted (Fig 4C), indicating that L2 formed a complex with endogenous KPNA2 through RanBP10.

Finally, we assessed whether Ran would be involved in the interaction of L2 and the RanBP10/KPNA2 complex. Ran is a Ras-related GTPase that plays fundamental roles in both interphase and mitosis [39]. Among other functions, it is responsible for regulating importin activities, MT-dependent transporter functions, and spindle formation [60–62]. Each function requires Ran-GTP hydrolysis, which is regulated by guanine nucleotide exchange factors (GEFs) and Ran-GTPase-activating proteins (GAPs) that promote Ran-GTP and Ran-GDP respectively. The so-called Ran-binding proteins (RanBP) often exert GEF or GAP functions [63–65]. Interestingly, Ran did not interact with L2-3xHA or HA-RanBP10 (Fig 4A) failing to support that the interaction between L2 and RanBP10/KPNA2 was a Ran-dependent process.

### KPNA2 facilitates HPV16 infectivity and L2 tethering

**Fig 5:**
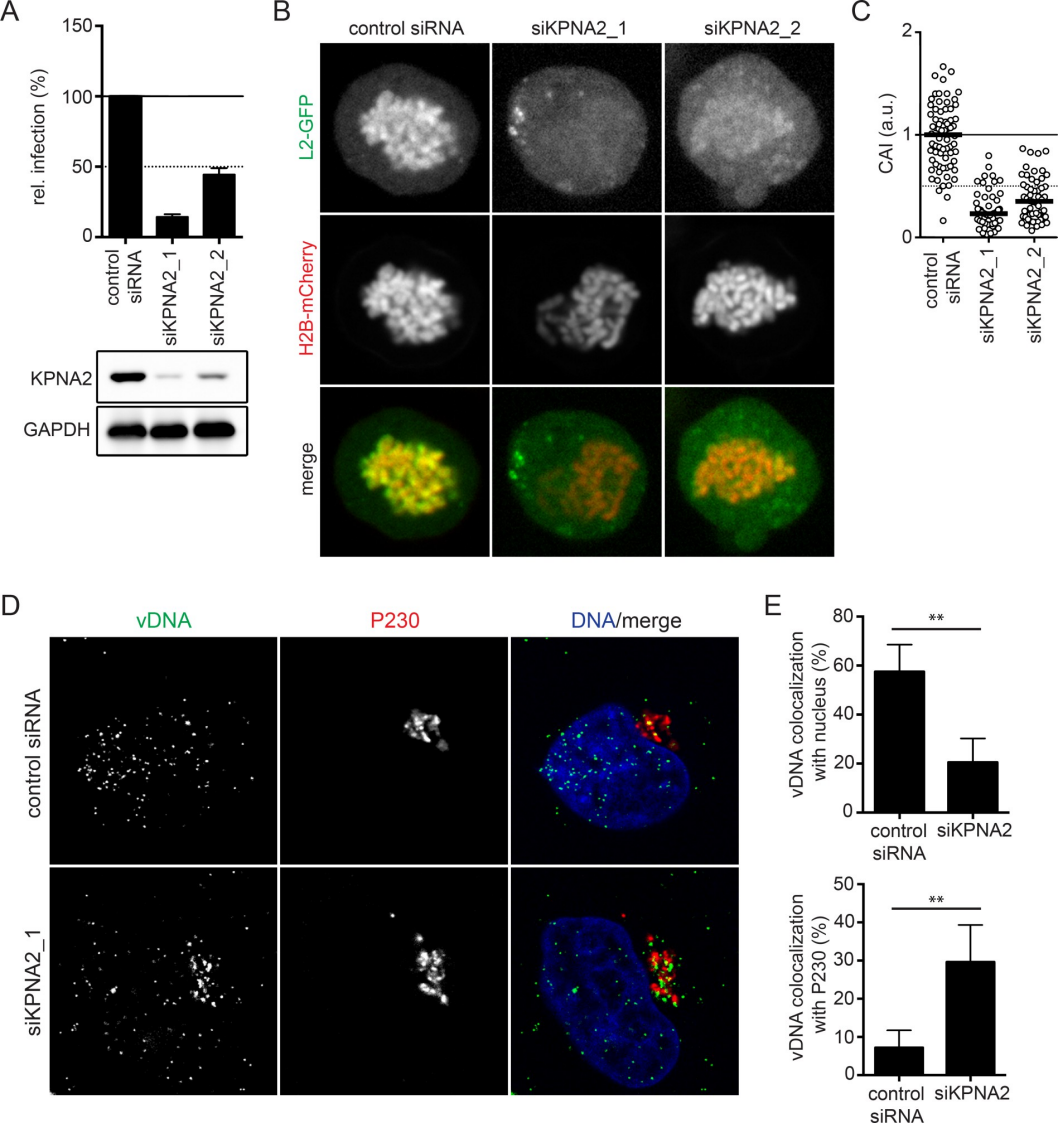
KPNA2 facilitated HPV16 infection and L2/vDNA nuclear import. **(A)** RNAi of KPNA2 by two individual siRNAs was followed by HPV16-PsV infection. Infectivity was scored based on cells expressing GFP over total cells. The infectivity was normalized to control siRNA transfected cells as relative (rel.) infection. Error bars indicate the SD of three independent experiments. **(B)** A L2 chromosomal association assay after KPNA2 depletion in HeLa Kyoto_H2B-mCherry_L2-GFP cells was performed as in Fig 2B. **(C)** CAI of panel B. At least 50 cells were analyzed in three independent experiments, and the median was indicated by the black bar. **(D)** HPV16 vDNA localization upon RNAi of KPNA2. HeLa cells were depleted of KPNA2 by siRNA transfection, followed by EdU-labelled HPV16 infection for 20 hours. P230 and Hoechst-33258 were stained to indicate TGN and nucleus, respectively. Confocal images depict median slices. **(E)** Co-localization of vDNA with the nucleus or P230 was quantified using IMARIS. At least 40 cells were analyzed in three independent experiments. Error bars indicate the SD.

To evaluate whether KPNA2 would functionally contribute to the role of RanBP10 in L2/vDNA nuclear delivery as a RanBP10/KPNA2 complex, we first assessed whether KPNA2 facilitated HPV16 infection. Upon individual depletion of KPNA2 by two siRNA, HPV16 infectivity was decreased by more than 50% establishing a functional role during infection (Fig 5A). Next, we assessed whether KPNA2 was responsible for L2/vDNA nuclear import. In our chromosomal association assay, L2 localization to mitotic chromatin was reduced by about 50–75% in KPNA2 depleted cells as compared to control cells similar to the reduction in infectivity (Fig 5B and 5C). These data indicated that the reduction in HPV16 infectivity was likely due to the failure in nuclear import of L2 upon knockdown of KPNA2. To further assess whether KPNA2 also contributed to the nuclear import of HPV16 in incoming virions, we employed EdU-labeled HPV16 PsVs to infect KPNA2-depleted cells. In control siRNA-treated cells, 57% and 7% of incoming vDNA localized to nucleus and Golgi, respectively. Upon knockdown of KPNA2, the localization of vDNA to the nucleus decreased to 20%, whereas localization to the Golgi increased to 30% (Fig 5D and 5E). Notably, the incoming vDNA was still able to reach the Golgi upon knockdown of KPNA2, suggesting that the early steps of HPV16 entry were not significantly affected. Taken together, these results were in line with the notion that KPNA2 functionally contributed to the transport of the L2/vDNA complex to mitotic chromatin through interaction with RanBP10 and L2.

### Dynein functions are important for the nuclear import of HPV16

**Fig 6:**
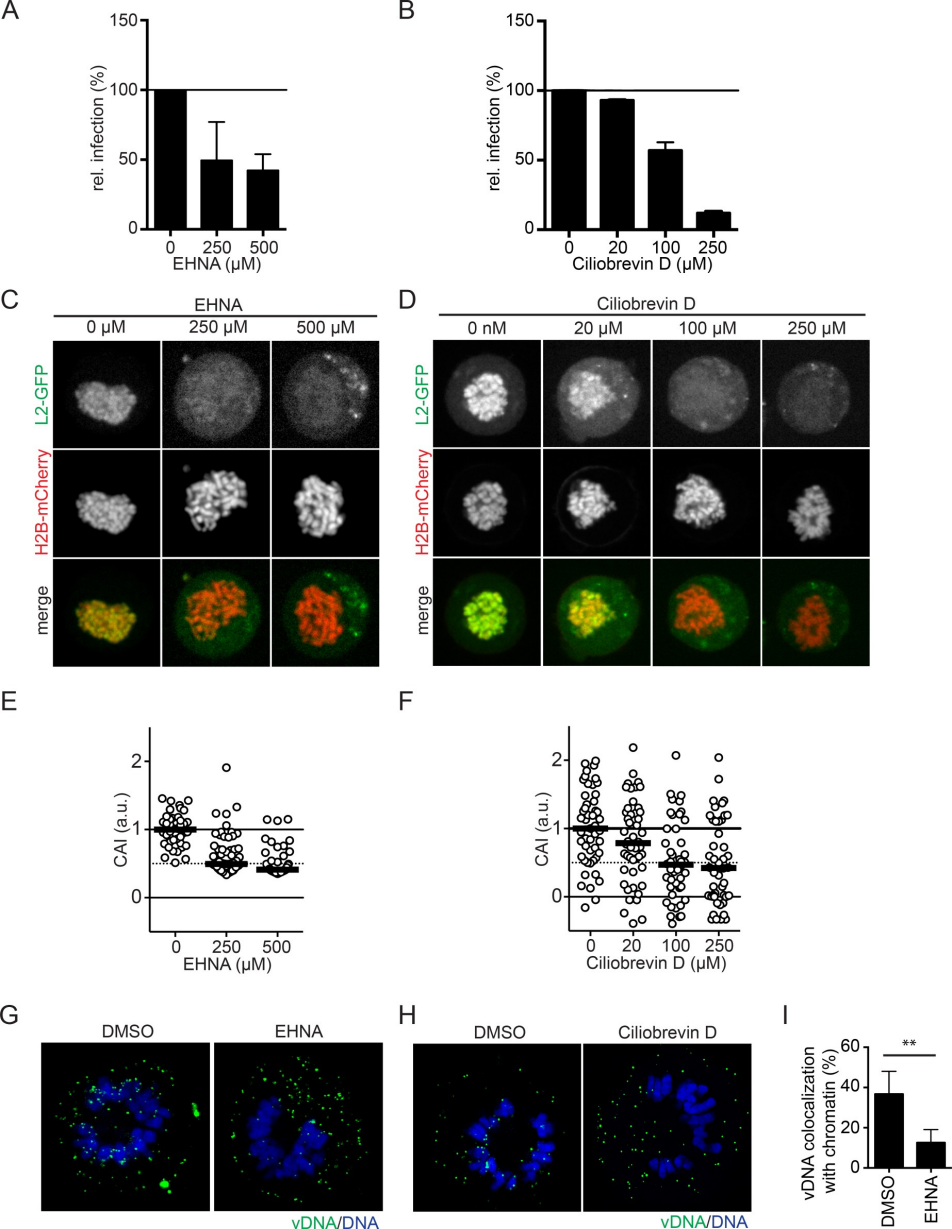
Dynein function was crucial for HPV16 infection and L2 tethering. EHNA or ciliobrevin D was used to inhibit dynein-mediated transport. HeLa Kyoto cells were treated with EHNA **(A)** or ciliobrevin D **(B)** one hour prior to and during HPV16 infection. The infectivity was scored based on the percentage of the cells expressing GFP with flow cytometry. The infectivity was normalized to DMSO-treated cells as relative infection. **(C)(D)** HeLa Kyoto_H2B-mCherry_L2-GFP cells were treated with EHNA **(C)** or ciliobrevin D **(D)** and nocodazole (330 nM) for 16 hours. L2 chromosomal association assay was assessed as described in Fig 2B. Depicted are single confocal slices. **(E)** and **(F)**: Quantification of **(C)** and **(D),** respectively. The CAI was normalized and analyzed as in Fig 2C. At least 40 cells were analyzed in three independent experiments, the median was indicated by the black bar. **(G)(H)** HeLa Kyoto cells were infected with EdU-labelled HPV16 and arrested in prometaphase as in Fig 3C. EHNA **(G)** or ciliobrevin D **(H)** was used to inhibit dynein activities for 2 hours prior to mitotic onset. Mitotic cells were fixed and vDNA was stained with EdU Click-iT chemistry. Host DNA was stained with Hoechst-33258 to indicate mitotic chromosomes. Depicted are single confocal slices. **(I)** Quantification of **(G)** of co-localized vDNA on mitotic chromosomes using IMARIS. More than 35 cells were analyzed in three independent experiments, error bars indicate the SD.

**Fig 7:**
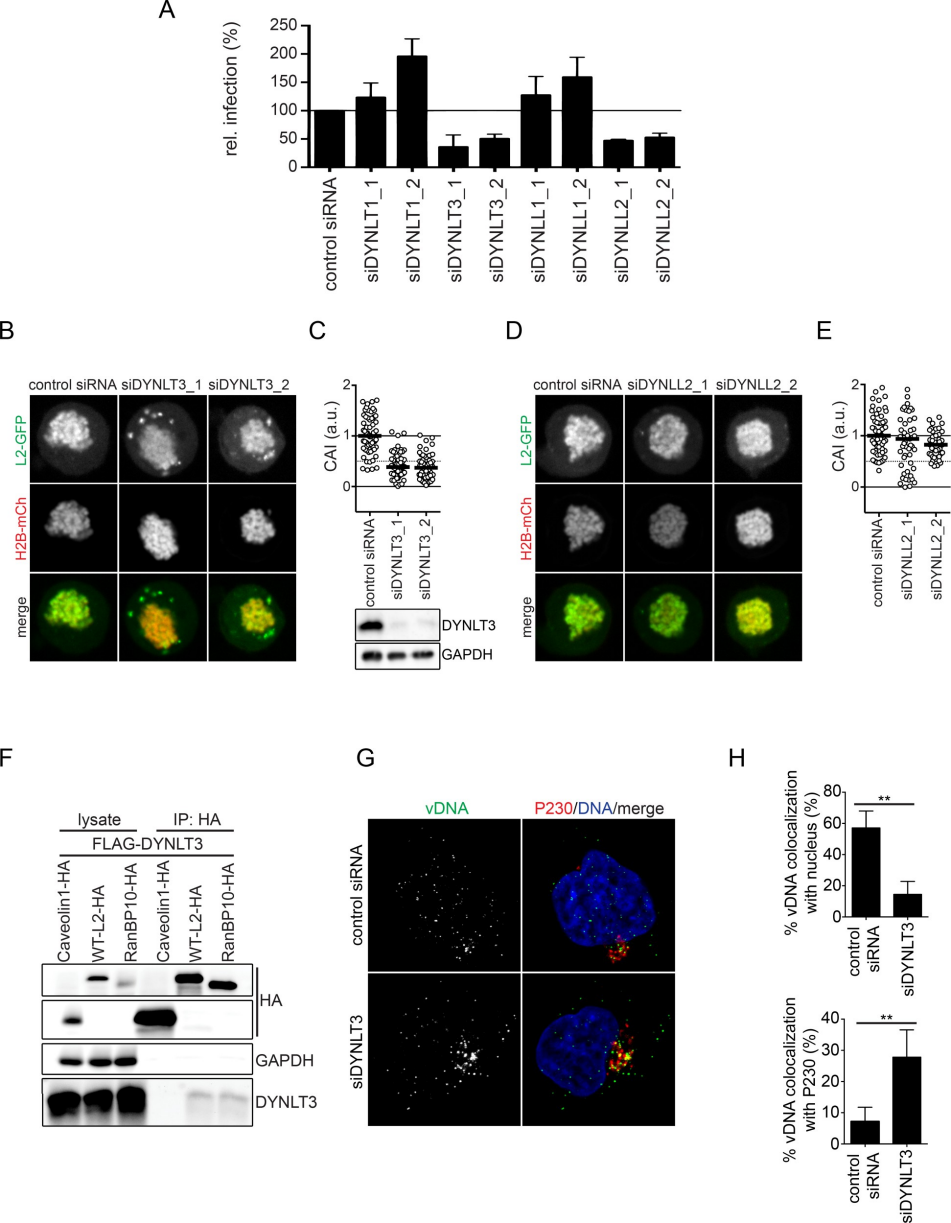
Dynein light chain DYNLT3 facilitates nuclear delivery of incoming HPV16. **(A)** RNAi of various dynein light chains by each two individual siRNAs as indicated in HeLa cells was followed by HPV16-PsV infection. Infectivity was scored based on cells expressing GFP over total cells. The infectivity was normalized to control siRNA transfected cells as relative (rel.) infection. Error bars indicate the SD of three independent experiments. **(B)(D)** A L2 chromosomal association assay after DYNLT3 **(B)** or DYNLL2 **(D)** depletion in HeLa Kyoto_H2B-mCherry_L2-GFP cells was performed as in Fig 2B. **(C)(E)** CAI of panels **(B)(D)**. At least 40 cells were analyzed in three independent experiments, and the median was indicated by the black bar. **(F)** Caveolin1-HA, L2-3xHA or HA-RanBP10 were expressed together with FLAG-DYNLT3 in HEK293 cells. Immunoprecipitation against HA was performed on cell lysates. Caveolin1-HA was used as a negative control of immunoprecipitation. HA, FLAG, and GAPDH were detected by Western Blotting after immunoprecipitation. **(G)** Knockdown of DYNLT3 in HeLa cells was followed by infection with EdU-labelled HPV16. Cells were fixed at 20 h.p.i., and stained with an anti-P230 antibody and Hoechst-33258 to indicate the TGN and nucleus, respectively. **(H)** Quantification of co-localized vDNA signals with nucleus or P230. At least 35 cells were analyzed in three independent experiments. The error bars indicate the SD.

Incoming vDNA from HPV16 virions has been localized to the MTOC in prometaphase [39, 40]. To migrate to the MTOC, L2/vDNA has to accomplish minus-end directed transport, which is typically mediated by cytosolic dynein motors. Previous studies support this hypothesis, as L2 is able to interact with dynein light chains [43, 44]. To assess the role of MTs and dynein motors in RanBP10/KPNA2-mediated subviral trafficking during nuclear import of HPV16, we resorted to pharmacological inhibition of microtubule polymerization with nocodazole or of dynein with erythro-9-3-(2-hydroxynonyl)adenine (EHNA)/ciliobrevin D, which inhibit dynein’s ATPase activity [66–68], as well as perturbation by RNAi. Nocodazole-induced, complete depolymerization of MTs during mitosis resulted in clearly reduced association of vDNA with mitotic chromatin (S4 Fig). However, reduced microtubule formation upon lower nocodazole concentrations of 330 nM used to block cells in prometaphase did not affect association of incoming vDNA with mitotic chromatin (S4 Fig). Upon EHNA or ciliobrevin D inhibition of dynein, HPV16 infectivity was reduced in a dose-dependent manner similar to Herpes simplex virus type 1 (HSV-1) infection that relies upon dynein-mediated transport during entry (Figs 6A, 6B, S5A and S5B, [66]). Probing for the role of dynein in nuclear import of HPV16, we next tested whether L2 association to mitotic chromatin was affected. EHNA or ciliobrevin D treatment significantly decreased L2 association with mitotic chromatin (Fig 6C–6F). To assess whether dynein activity played a direct role in L2/vDNA nuclear import, cells infected with EdU-labelled HPV16 were treated with EHNA or ciliobrevin D 1 hour prior to entering mitosis. The amount of incoming vDNA that localized to mitotic chromosomes upon EHNA or ciliobrevin D treatment was clearly reduced as compared to the control suggesting dynein played an important role in HPV16 nuclear import (Fig 6G–6I). To more specifically interfere with dynein function, we depleted cells of dynein light chains (DYNLT1, DYNLT3, DYNLL1, and DYNLL2) that link cargo via adaptor molecules to the core dynein motor [69–73], and tested whether HPV16 infectivity would be affected. RNAi of DYNLT3 and DYNLL2 reduced HPV16 infection by about 50%, whereas DYNLT1 and DYNLL1 did not decrease infectivity (Fig 7A). In confirmation, CRISPR/Cas9 knockout of DYNLL2 expression reduced HPV16 infectivity also by about 50% (S5C Fig). Thus, specific dynein light chains contributed to dynein-mediated transport during HPV16 infection. Next, we assessed whether dynein light chains would facilitate L2 association with mitotic chromatin. Upon silencing of DYNLT3, L2 associated about 50% less with mitotic chromatin, whereas DYNLL2 silencing displayed no significant reduction (Fig 7B–7E). This suggested that DYNLT3 facilitated transport of L2 during mitosis. To test, whether DYNLT3 may in fact associate with the L2/RanBP10/KPNA2 complex important for nuclear import, we resorted again to immunoprecipitation. In line with the formation of a transport complex, exogenously expressed DYNLT3 co-immunoprecipitated with L2 or RanBP10 but not Caveolin-1 suggesting a physical interaction (Fig 7F). Finally, the contribution of DYNLT3 to nuclear import was assessed. Similar to RanBP10 and KPNA2 depletion, RNAi DYNLT3 reverted the localization of vDNA from nucleus to Golgi (Fig 7G and 7H). Moreover, DYNLT3-depletion also led to a decrease of vDNA localization to mitotic chromatin (S6A Fig) but did not accumulate vDNA in endosomal compartments (S6B and S6C Figs). This indicated that in DYNLT3-depleted cells HPV16 transport from the Golgi to mitotic chromatin was impaired.

## Discussion

**Fig 8:**
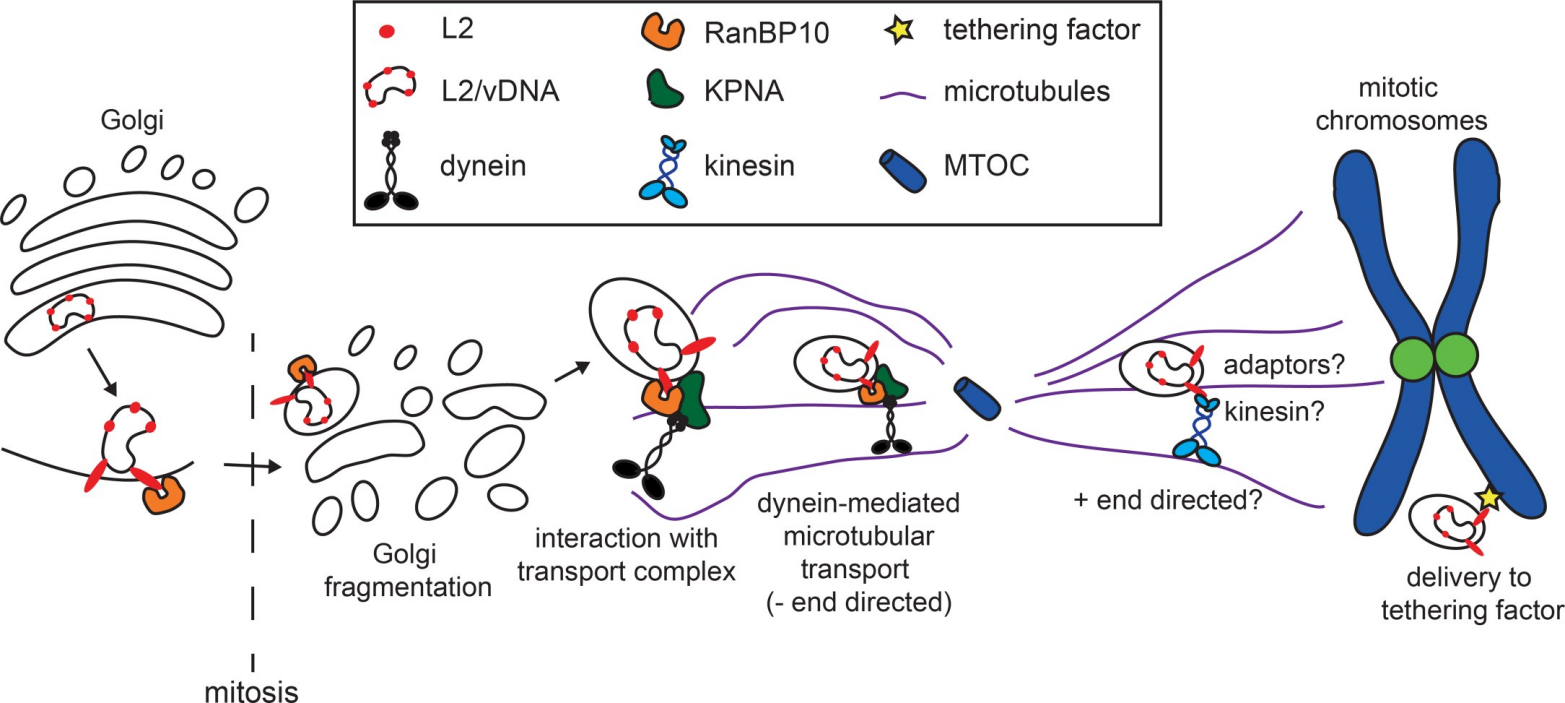
Working model of RanBP10-mediated subcellular transport during the nuclear import of HPV16. The L2 from incoming HPV16-PsV interacts with RanBP10, which interacts with cytosolic KPNA2 and thereby associates with MT-dependent motor proteins. The interaction forms a transport complex together with dynein motors that facilitates the subcellular trafficking of L2/vDNA complex. However, RanBP10 does not associate to mitotic chromosomes indicating a potential chromatin tethering factor still needs to be identified.

In this study, a novel cellular interaction partner of HPV16 L2, namely RanBP10, was identified that was important for the delivery of L2 and incoming viruses onto mitotic chromatin to thereby accomplish the nuclear import of vDNA. Mechanistically, RanBP10 linked L2, KPNA2, and DYNLT3 forming a complex that likely facilitated minus-end directed dynein-dependent transport of incoming L2/vDNA during mitosis (Fig 8).

RanBP10 physically interacted with L2 mainly in the perinuclear area, where nuclear import of HPV16 is initiated at the onset of mitosis [38, 41]. Upon depletion of RanBP10, the vDNA was found localized to the Golgi apparatus and failed to reach the nucleus indicating that early steps of cell entry were independent of RanBP10. In contrast, RanBP10 played an important role during nuclear import of vDNA. Similarly, overexpressed L2 associated about 50% less with mitotic chromatin upon RanBP10 depletion. Nuclear import of HPV16 during mitosis can be subdivided into four critical steps that are necessary for cell entry, and that are likely coordinated in time and space: (i) Golgi fragmentation and vesiculation of the subviral complex to be tethered to mitotic chromatin [39, 40]; (ii) penetration of L2 C-termini through the enclosing vesicular membrane [40]; (iii) transport of the vesicle containing the subviral complex towards mitotic chromosomes; and (iv) tethering of the subviral complex to mitotic chromatin to ensure incorporation into nascent nuclei [41].

Since proper Golgi fragmentation is crucial for progression through mitosis, and since it may contribute to generate a virus-containing vesicle for nuclear import, RanBP10 depletion might have affected these processes and thereby caused reduced infectivity and chromosomal association. Moreover, KPNA2 can directly bind to the Golgi matrix protein, GM130, during mitosis to aid Golgi disassembly [74]. However, neither mitosis nor Golgi fragmentation during mitosis (S3B Fig) were affected upon knockdown of RanBP10 or KPNA2. Thus, it is highly unlikely that RanBP10 or KPNA2 facilitated nuclear import through Golgi fragmentation and vesiculation.

It is also highly unlikely that RanBP10 is responsible for tethering L2 or incoming virus to mitotic chromatin. If this were to be the case, one would expect a localization of RanBP10 together with L2 or incoming virions to mitotic chromatin. However, no significant co-localization was observed.

Recent descriptive work suggests that L2/vDNA transport during nuclear import may occur by MT-dependent transport [39, 40]. MT-dependent transport of cargo is facilitated by motor proteins. Cargo is most often linked to the motor proteins by adaptor proteins (reviewed in [75]). Dyneins or kinesins then move cargo either towards the minus or plus end of MTs, respectively (reviewed in [76]). During mitosis, the minus end localizes to the MTOC/centrosomes, whereas the plus ends are localized at the plasma membrane or mitotic chromosomes [77] (reviewed in [78]). Evidence suggests that the vDNA transiently localizes to the MTOC during mitosis [40]. Moreover, it has been shown that L2 interacts with dynein light chains [43, 44]. Thus, it is reasonable to assume MT-dependent transport of HPV16 also during mitosis. Our data confirms such a MT-dependent transport step during mitosis. This transport likely involves both dynein- and kinesin-dependent transport. Dynein-mediated minus end-directed transport would be required for vesicles containing the vDNA towards the MTOC, whereas kinesin-mediated plus end-directed transport would direct the vDNA from the MTOC towards mitotic chromatin.

Dynein-dependent processes clearly contributed to nuclear import of HPV16: First, dynein motor function as well as dynein light chain DYNLT3 were required for nuclear import of HPV16 and association of L2 with mitotic chromatin. Second, importin subunits interact with dynein light chains to transport cargo toward the minus end of MT during mitosis [56–58]. Our data demonstrates for the first time a functional complex of cellular KPNA2 and RanBP10, which interacted with L2 through RanBP10. While KPNA2 has importin functions for shuttling cargo through the NPC, any interaction with incoming L2 during interphase would not result in nuclear import, because the vDNA locates to the Golgi during this phase [27, 40]. Since both KPNA2 and RanBP10 facilitated nuclear import and localization of L2 to mitotic chromatin phenocopying the effect of dynein motor perturbation and depletion of DYNLT3, RanBP10/KPNA2 rather constitutes an adaptor complex to facilitate dynein-dependent process during mitosis. This hypothesis is supported by our data indicating a physical interaction of L2 and RanBP10 with DYNLT3. It is interesting to note that engagement of only one of the two dynein light chains known to specifically interact with L2 [43] promotes nuclear import suggesting that L2 may engage different dynein light chains and perhaps adaptors during cell entry–likely tailored for the specific transport step, such as endosomal and retrograde trafficking to the Golgi [30, 36].

So where does the vDNA reside upon perturbation of the complex? Depletion of DNYLT3 fails to arrest vDNA in endosomal compartments in line with unperturbed intracellular trafficking to the Golgi. Since our data indicates that RanBP10 depletion does not impair Golgi disassembly, the vDNA likely resides in a Golgi-derived vesicle. In support, upon Golgi reformation after mitosis the vDNA co-localizes with Golgi markers suggesting that the vDNA containing vesicles are able to fuse back with the Golgi, if nuclear import during mitosis fails.

It is important to note that the L2/RanBP10/KPNA2/DYNLT3 complex would only support virus transport to MTOCs/centrosomes. Hence, kinesin-mediated, plus end-directed transport from the MTOC to mitotic chromatin is likely to be required for successful HPV16 entry. In support, HPV16 entry was not abrogated upon efficient RanBP10 depletion or dynein-inhibition and about 50% of the infectivity remained (Figs S1B, 6A and 6B). Thus, about 50% of incoming HPV16 may be able to engage kinesin motors during mitosis independent of dynein-dependent transport. In line with this hypothesis, some vDNA still reached mitotic chromatin in RanBP10-depleted cells, while at the same time accumulations of vDNA close to the plasma membrane and around the MTOC appeared (Fig 3A–3E). This is reminiscent of kinesin-mediated transport of cargo upon dynein inhibition: vDNA-containing vesicles not engaging kinesins would remain localized to the MTOC area failing nuclear delivery. vDNA-containing vesicles that engage kinesins would end up at two different locations: Vesicles located closer to astral MTs would be transported closer to the plasma membrane and would fail to deliver vDNA to mitotic chromatin, whereas those in proximity to polar MTs would be able to engage kinesins, associate with mitotic chromatin for nuclear entry and cause infection.

Although our mass spectrometry data indicated an interaction of L2 with the kinesin KIF20B (S1 Table), it is unlikely that plus-end directed transport is facilitated by this particular kinesin, mostly because KIF20B facilitates microtubule sliding during mitosis but not transport [79, 80]. In addition, incoming vDNA was still imported into the nucleus upon KIF20B silencing. Based on the fact that 14 different kinesin families exist (reviewed in [81]), it will be important to systematically analyze which kinesins may be involved in plus end-directed transport of HPV16.

While it is likely that a subviral complex that also seems to be enclosed in a vesicle would require some form of active transport to reach mitotic chromatin, one might question if ectopically expressed L2-GFP would not be able to reach mitotic chromatin by diffusion rather than active transport as indicated by our data. Since the effects on L2-GFP chromatin association upon perturbation of the RanBP10/KPNA2/L2 complex are milder than on vDNA association, it is likely that at least some L2-GFP is able to reach mitotic chromatin by diffusion while being more efficient in reaching mitotic chromatin with an active transport system in place.

In summary, our work identified the first cellular protein complex that facilitates the nuclear import of HPV16 during mitosis. Moreover, our findings on the novel interaction between RanBP10 and KPNA2 describe a novel cellular adaptor complex for intracellular dynein-mediated transport that is hijacked by a virus, and that potentially also has a role in cellular physiology. While its cellular function remains yet elusive, our findings expand our knowledge on an understudied cellular protein.

## Materials and methods

### Cell lines, plasmids, reagents, antibodies, and viruses

HeLa Kyoto and HeLa H2B-mCherry cells were kindly provided by L. Pelkmans, (University of Zurich, Switzerland) and D. Gerlich (IMB, Vienna, Austria), respectively. HeLa Kyoto H2B-mCherry_L2-GFP cells were generated in our lab by transfection of pL2-EGFP and clonal selection with Hygromycin B. HEK293 were from ATCC. HEK293TT cells and pL2-EGFP were kindly provided by C. Buck (NIH, Bethesda, USA). HeLa cells with a DYNLL2 CRISPR/Cas9 knockout in exon 2 (ab265158) were purchased from Abcam. Expression plasmids of truncated L2 (N-terminal NLS deleted L2-GFP, N- and C-terminal NLS deleted L2-GFP) are described in [41]. pL2-3xHA and pL2-3xFLAG were a kind gift from L. Florin (University of Mainz, Germany) [82, 83]. The FLAG-DYNLT3 expression plasmid was purchased from Sino Biological (HG20799-CF). pL2(RTR313EEE)-3xHA was produced by site-directed mutagenesis using pL2-3xHA as described in [41]. pMXs-HA-RanBP10 was kindly provided by Y. Kawaguchi, University of Tokyo [84]. The EcoRI (5´) and NotI (3´) site on pMXs-HA-RanBP10 was digested to obtain the HA-RanBP10 coding sequence, which was then introduced into pcDNA3 through EcoRI (5´) and NotI (3´) site by standard cloning procedures. Aphidicolin, nocodazole, ciliobrevin D, and EHNA were from Sigma-Aldrich. MitoTracker Red was from Thermo Fisher. RO-3306 was purchased from Selleckchem. RanBP10 (#21107-1-AP) and GAPDH (#10494-1-AP) polyclonal antibodies were from Proteintech [85]. The anti-HA antibody (HA.11) was from BioLegend. KPNA2 (B-9) and Ran (A-7) were from Santa Cruz Biotechnology. Alpha-tubulin (DM1A, T6199) and anti-FLAG antibody (M2) were from Sigma-Aldrich. Anti-GFP antibody (4B10) was from Cell Signaling. P230 (#611280) was obtained from BD Transduction Laboratories. The anti-DYNLT3 antibody was from Abcam (ab121209). HPV16-PsVs with or without 5-ethynyl-2’-deoxyuridine (EdU)-labelled GFP reporter plasmid were generated using p16Shell and pCleno-GFP as previously described [41].

### RNA interference

The RNA interference was performed in either 96- or 12-well plates with Lipofectamine RNAiMAX transfection reagent (Thermo Fisher). In 96-well plates, 1500 or 3000 HeLa cells were transfected respectively with 10 nM or 20 nM of siRNA for 48 hours. In 12-wells, 3x10^4^ HeLa cells were transfected with 20 nM siRNA for 48 hours. Alternatively, for RanBP10, cells were transfected for 24 hours with the indicated amounts of siRNA, and then transfected again with the same amount of siRNA for further 24h. All the siRNAs were obtained from Qiagen. The siRNAs used in this study are: RanBP10_1 (SI02644355), siRanBP10_2 (SI02644362), siKPNA2_1 (SI05151482), siKPNA2_2 (SI05151489), siDYNLT1_1 (SI05010558), siDYNLT1_2 (SI05010565), siDYNLT3_1 (SI04286303), siDYNLT3_2 (SI04374503), siDYNLL1_1 (SI04238780), siDYNLL1_2 (SI04323921), siDYNLL2_1 (SI04331698), siDYNLL2_2 (SI05076414), KIF20B_1 (SI05108796), KIF20B_2 (SI05108803), KIF20B_3 (SI02780855). As controls, AllStar Negative Control siRNA (SI03650318) and AllStars Cell Death siRNA (SI04381048) were used. The knockdown efficiencies were verified on the mRNA level with RT-qPCR (S7 Fig) and/or on the protein level by Western blotting.

### HPV16 PsV infection

In brief, cells were infected with 6 ng of HPV16-PsV for 48 hours in 12-well plates to result in 20–30% infected cells. To monitor the incoming vDNA localization, synchronized cells were infected with 20 ng of EdU-labelled HPV16 for 20 hours. Cells were fixed with 4% paraformaldehyde and the infectivity was scored by flow cytometry or microscopy as previously described [38, 41].

### L2 chromosomal association assay

The L2 chromosomal association assay was carried out as previously described [41]. Briefly, 3000 HeLa cells stably expressing H2B-mCherry and L2-GFP were transfected with 20 nM siRNA in 96-well plates for 48 hours. At 48 hours post transfection, cells were synchronized with nocodazole (330 nM) for 16 hours. Mitotic cells were fixed with 4% paraformaldehyde, and Alexa Fluor 647 Phalloidin served as a counterstain. Single slice images were acquired with Zeiss Axio Observer.Z1 spinning disc microscope (40x PlanApo oil immersion objective) and analyzed with CellProfiler. The chromatin regions were defined by the H2B-mCherry signals. The cytosol regions were defined by phalloidin staining. The chromosomal association index (CAI) was given by the ratio of the mean intensity of GFP in the chromatin region over the cytosol area.

### Immunofluorescence staining and localization of incoming vDNA

To monitor the incoming vDNA localization, cells were infected with EdU-labelled HPV16 [41] for 20 hours. To enrich for mitotic cells, cells were synchronized with 9 μM RO-3306 (Selleckchem) in G2/M, and fresh medium was used to release the cells into mitosis for 30 minutes. Cells were fixed with 4% paraformaldehyde, and then the Click-iT EdU AF488 imaging kit (Thermo Fisher) was used to visualize vDNA. To determine vDNA localizations, endogenous RanBP10 (Proteintech), alpha-tubulin (Sigma T6199), pericentrin (Abcam ab4448), and P230 (BD Biosciences) antibodies in 3% BSA were used to perform immunofluorescence staining. The nuclei were stained with Hoechst-33258. Image stacks were acquired using a LSM800 (63x objective) confocal microscope. The vDNA co-localization was analyzed with IMARIS Coloc function. The amount of vDNA locating on mitotic chromosomes, MT, or Golgi apparatus were quantified by the co-localization of vDNA signals in voxels with Hoechst-33258, alpha-tubulin, or P230, respectively. Co-localized vDNA signals (voxels) were divided by the total vDNA signals and shown in percentage. Around 15 random cells were imaged and analyzed in each individual experiment, and quantified for at least three independent experiments. Statistical significance of co-localized vDNA signals were then assessed by student´s t-test (n.s. P>0.05, * P < 0.05, ** P < 0.01, *** P < 0.001). Proximity to the MTOC was determined by thresholding of the pericentrin signal, and extending a 2 μm radius around the signal. Similarly, the plasma membrane area was determined by thresholding the cells border signal, and a 2 μm margin extending into the cells cell margin was determined. The vDNA signals were determined within these margins, and normalized to the control.

### SRRF analysis of HPV16 vDNA on mitotic microtubules

Super-resolution and radial fluctuations (SRRF) was performed following published procedures [86]. In brief, 100 images of the same cell and the same focal plane were acquired under signal fluctuations conditions using a spinning disk confocal microscope (Zeiss AxioObserver.Z1, equipped with a Yokogawa CSU22 spinning disk unit, 100x oil immersion objective, N.A. 1,45, Visitron). A theoretical point-spread function was determined and image were deconvolved using Deconvolution Lab 3D plug-in for ImageJ/Fiji [87], by applying the Richardson-Lucy algorithm with 10 iterations. Deconvolved vDNA and MTs stacked images were then imported to NanoJ-SRRF plug-in and SRRF analysis was performed (ring radius: 0.5, radiality magnification: 5, axes in ring: 8). Super-resolved vDNA and MTs images were converted into 8-bit images, thresholded in ImageJ/Fiji (vDNA: Otsu method, MTs: Li method) and then imported into the JaCOP plug-in for ImageJ/Fiji [88]. The particle-based colocalization algorithm was applied to detect vDNA signal overlapping with MTs signal. A total of 15 random cells for three independent experiments were analyzed and quantified.

### Immunoprecipitation

5x10^6^ HEK293 cells were seeded one day prior to transfection in 10 cm petri dishes. Cells were transfected with 5 to 10 μg of plasmids/Lipofectamine2000 (Invitrogen) for 24 hours. Cells were then synchronized with either aphidicolin (3 μM) or nocodazole (330 nM) for 16 hours. For siRNA knockdown experiments, HeLa cells were reverse-transfected with siRNA in 12-well plates for 48 hours as described above, followed by overexpression of L2-3xHA for 24 hours. Cells were harvested and lysed with IP lysis buffer (150 mM NaCl,1% NP-40, 50 mM Tris-HCl, 50 mM NaF, pH 7.4) and pre-cleared with protein G agarose beads (Sigma). 10% of the samples were taken as input, the rests were incubated with primary antibodies (anti-HA, HA.11, BioLegend, or anti-FLAG M2, Invitrogen) and protein G agarose beads. Agarose beads were washed sequentially with wash buffers, and the samples were boiled at 95°C with SDS-sample buffer. Proteins of interest were then detected by Western blotting.

### Mass spectrometry

The immunoprecipitation assay was performed as described above. For mass-spectrometry analysis, precipitated proteins were size fractionated by denaturing SDS-PAGE using 4–12% Bis-Tris gradient gels (life technologies), and in-gel digested with trypsin. Extracted and desalted peptides of each fraction were then subjected to liquid chromatography coupled tandem mass spectrometry using an EASY-nLC coupled to an Orbitrap Velos mass spectrometer via a Nanospray Flex ion source which harboured a C18 reversed phase capillary column with integrated emitter tip (360μm OD x 75μm ID x 150mm length; in house packed with Reprosil Pur c18-AQ; 3.0μm, Dr. Maisch). Peptides were separated at a flow rate of 250 nl/min using a multistep gradient, running from 2–7% Buffer B (0.1% formic acid, 80% acetonitrile) in 5 mins, from 7–35% B in 90min, from 35–60% B in 20 min. The column was flushed at 98% B for additional 12 mins before re-equilibration at starting conditions (Buffer A: 0.1% formic acid). The Orbitrap Velos mass spectrometer was operated in data-dependent acquisition mode (spray voltage 2.3 kV) collecting collision induced MS/MS spectra from the fifteen most intense peaks in the MS (LTQ-FT full scans from *m*/*z* 300 to *m*/*z* 1650; resolution *r* = 60,000; LTQ isolation and fragmentation at a target value of 10000). Dynamic exclusion of previously identified peptides was activated (list size 500, exclusion duration 90s). Data were recorded with Xcalibur software (Thermo Scientific). MS data were analyzed using the label-free quantification algorithm embedded in the MaxQuant v1.5.3.12 proteomics software [89]. Identification of peptides and proteins was enabled by the built-in Andromeda search engine by querying the concatenated forward and reverse human Uniprot database (UP000005640_9606.fasta) including common lab contaminants. Allowed initial mass deviations were set to 0.5 Da and 20 ppm, respectively, in the search for precursor and fragment ions. Trypsin with full enzyme specificity and only peptides with a minimum length of 7 amino acids was selected. A maximum of two missed cleavages was allowed; the ‘match between runs’ option was turned on. Carbamidomethylation (Cys) was set as fixed modification, while Oxidation (Met) and N—acetylation at the protein N-terminus were defined as variable modifications. For peptide and protein identifications a minimum false discovery rate (FDR) of 1% was required. The results from the MaxQuant analysis were then bioinformatically processed using Perseus. Reverse hits from the search were subsequently removed from the list of identified proteins, as well as common lab contaminants and proteins that were only identified by a single modified peptide and that were not detected with at least 1 unique peptide.

Replicates (n = 3) of each condition were annotated to two separate groups (wild-type and mutant). Proteins containing at least two valid LFQ values in at least one group were then further selected. To handle the problem of missing (LFQ) intensity values following log_2_ transformation, NaN entries in the list were replaced by imputation with values from the far left of a normal distribution of all LFQ values determined in the entire data set (down shift 1.8, width = 0.3). Statistical significance of differential expression was then assessed by a two sample Student´s t-test (p = 0.05) comparing wild-type and mutant groups.

## Supporting information

S1 Data Excel spreadsheet containing numerical values used to generate graphs statistical analysis for Figs 1F, 2A, 2C, 3B, 3D, 3E, 3G, 5A, 5C, 5E, 6A, 6B, 6E, 6F, 6I, 7A, 7C, 7E, 7H, S1B, S2B, S4B, S5A, S5B, S5C, S6C and S7. (XLSX)

S1 Table List of potential interactors of L2 during mitosis. The label-free semi-quantitative mass spectrometry and statistical analysis were performed as described in material and methods. Columns: iBAQ (intensity based absolute quantification) represents the total intensities divided by the number of theoretical peptides, i.e. a value proportional to the molar quantities of a protein, thereby providing a rough estimate of the abundance of a protein in each sample. MS/MS count references the total sequenced events for a peptide. Score refers to the Andromeda score for the best associated MS/MS spectrum. The 47 potential cellular interactors of L2 were individually depleted in HeLa cells with three independent siRNA followed by HPV16 infectivity assays or L2 CAA with two siRNAs in HeLa Kyoto_H2B-mCherry_L2-GFP cells, with (—) at least 50% reduction, (┼) at least 50% increase, ⏀: no significant phenotype, n.d.: not determined. Candidates exhibited 50% reduction in both assays for at least two siRNAs.
(TIFF)

S1 Fig Impact of RanBP10 on L2 interaction, HPV16 infection, and L2 localization. **(A)** Caveolin1-HA, L2-WT-3x-HA, and L2-RTR313EEE-3xHA were individually expressed in mitotic HEK293 cells to perform immunoprecipitation assay. Caveolin1-HA was used as a negative control. Endogenous RanBP10 and HA-tag were detected by western blotting. **(B)** RNAi of RanBP10 in HeLa cells using different amounts of siRNA was followed by HPV16-PsV infection for 48 hours. Infectivity was scored by flow cytometry based on the percentage of the cells expressing GFP. The infectivity was normalized to control siRNA transfected cells and depicted as relative (rel.) infection. The protein expression level of RanP10 upon siRNA knockdown was analyzed by Western Blotting. **(C)** HeLa cells were co-transfected with HA-RanBP10 and/or L2-GFP expressing plasmids. Nucleus was stained with Hoechst-33258. Images were acquired with LSM800 in 700 nm single slices. Images were presented in single median slices.
(TIF)

S2 Fig SRRF microscopy analysis of vDNA/MT association. **(A)** Co-localization of incoming vDNA and MTs. HeLa cells were infected with EdU-labelled HPV16 and arrested in mitosis. Cells were stained for vDNA and alpha-tubulin (MTs). Cells were analyzed by SRRF microscopy as described in material and methods. **(B)** Quantification of co-localized vDNA with MTs in the super-resolved images. Data represents the average of three independent experiments ±SD.
(TIF)

S3 Fig Golgi fragmentation and vesiculation were not affected upon knockdown of RanBP10 and KPNA2. **(A)** HeLa cells during mitosis stained for endogenous RanBP10, KPNA2, and DNA. **(B)** HeLa cells with RanBP10 or KPNA2 depletion via RNAi were infected with EdU-labelled HPV16. After 20 h.p.i, cells synchronized in mitosis were fixed and stained with anti-Giantin antibody and Hoechst-33258 to visualize Golgi and mitotic chromosomes. The incoming vDNA labelled with EdU was detected by EdU Click-iT chemistry. Images were acquired by confocal microscopy. Images represent single median slices.
(TIF)

S4 Fig Impact of MT depolymerization on delivery of vDNA to mitotic chromatin. Nocodazole at the different indicated concentrations was used to interfere with MT polymerization. **(A)** HeLa Kyoto cells were infected with EdU-labelled HPV16 treated with nocodazole at the indicated concentrations two hours prior to mitosis in synchronized cells. Mitotic cells were fixed and vDNA was stained with EdU-Click-iT chemistry. Host DNA was stained with Hoechst-33258 to indicate mitotic chromosomes. Depicted are single confocal slices. **(B)** Quantification of co-localized vDNA signals with mitotic chromatin upon nocodazole treatments. At least 35 cells were analyzed in three independent experiments. The error bars indicate the SD. n.s.: not significant. **(C)** HeLa Kyoto cells were treated with nocodazole at the indicated concentrations two hours prior to mitosis in synchronized cells. Mitotic cells were fixed and MTs were stained with an alpha-tubulin antibody. Host DNA was stained with Hoechst-33258 to indicate mitotic chromosomes. Depicted are single confocal slices.
(TIF)

S5 Fig Interference with dynein function affects HSV-1 and HPV16 infectivity. EHNA or ciliobrevin D was used to inhibit dynein-mediated transport. HeLa Kyoto cells were treated with EHNA **(A)** or ciliobrevin D **(B)** one hour prior to and during HSV-1-GFP infection. The infectivity was scored based on the percentage of the cells expressing GFP with flow cytometry. The infectivity was normalized to DMSO-treated cells as relative infection. **(C)** Infection of WT and DYNLL2 CRISPR/Cas9 knockout (KO) HeLa cells. The infectivity was scored based on the percentage of the cells expressing GFP with flow cytometry. The infectivity was normalized to DMSO treated cells as relative infection.
(TIF)

S6 Fig RNAi of DYNLT3 interferes with mitotic chromatin association not through stalling HPV16 in endosomal compartments. **(A)** HeLa Kyoto cells treated with siRNA against DYNLT3 or control were infected with EdU-labelled HPV16 and arrested in pro-metaphase as in Fig 2B. Mitotic cells were fixed and vDNA was stained with EdU-Click chemistry. Host DNA was stained with Hoechst-33258 to indicate mitotic chromosomes. Depicted are single confocal slices. **(B)** HeLa Kyoto cells treated with siRNA against DYNLT3 or control were infected with EdU-labelled HPV16 for 20h. Cells were fixed and vDNA was stained with EdU Click-iT chemistry, and endosomes with an antibody against LAMP1. Host DNA was stained with Hoechst-33258 to indicate cell nuclei. Depicted are single confocal slices. **(C)** Quantification of co-localized vDNA with cell nuclei or LAMP1 using IMARIS. More than 35 cells were analyzed in three independent experiments, error bars indicate the SD.
(TIF)

S7 Fig Efficiency of RNA interference in mRNA level. RNAi was performed on HeLa cells as described in materials and methods. The total RNA was extracted with RNA extraction kit from Qiagen (RNeasy kit), and reverse transcription was performed with poly-T primer. Real-time PCR was used to detect the mRNA level of RNAi target protein, as well as GAPDH for normalization. All samples were normalized to control siRNA transfected cells to obtain relative mRNA expression levels. RNAi experiments were performed individually three times.
(TIF)

## References

[1] Modis Y, Trus BL, Harrison SC. Atomic model of the papillomavirus capsid. EMBO J. 2002;21: 4754–4762. 10.1093/emboj/cdf494 12234916 PMC126290

[2] Buck CB, Cheng N, Thompson CD, Lowy DR, Steven AC, Schiller JT, et al. Arrangement of L2 within the Papillomavirus Capsid. J Virol. 2008;82: 5190–5197. 10.1128/JVI.02726-07 18367526 PMC2395198

[3] Conway MJ, Meyers C. Replication and assembly of human papillomaviruses. J Dent Res. 2009;88: 307–317. 10.1177/0022034509333446 19407149 PMC3317948

[4] Finnen RL, Erickson KD, Chen XS, Garcea RL. Interactions between Papillomavirus L1 and L2 Capsid Proteins. J Virol. 2003;77: 4818–4826. 10.1128/jvi.77.8.4818-4826.2003 12663788 PMC152166

[5] Zheng ZM, Baker CC. Papillomavirus genome structure, expression, and post-transcriptional regulation. Front Biosci. 2006;11: 2286–2302. 10.2741/1971 16720315 PMC1472295

[6] Doorbar J, Quint W, Banks L, Bravo IG, Stoler M, Broker TR, et al. The biology and life-cycle of human papillomaviruses. Vaccine. 2012;30: F55–F70. 10.1016/j.vaccine.2012.06.083 23199966

[7] Egawa N, Egawa K, Griffin H, Doorbar J. Human papillomaviruses; Epithelial tropisms, and the development of neoplasia. Viruses. 2015;7: 3863–3890. 10.3390/v7072802 26193301 PMC4517131

[8] Taichman LB, Reilly SS, LaPorta RF. The role of keratinocyte differentiation in the expression of epitheliotropic viruses. Journal of Investigative Dermatology. 1983. pp. S137–S140. 10.1111/1523-1747.ep12540909 6190958

[9] Yeo-Teh NSL, Ito Y, Jha S. High-risk human papillomaviral oncogenes E6 and E7 target key cellular pathways to achieve oncogenesis. Int J Mol Sci. 2018;19. 10.3390/ijms19061706 29890655 PMC6032416

[10] Zur Hausen H. Papillomaviruses and cancer: From basic studies to clinical application. Nat Rev Cancer. 2002;2: 342–350. 10.1038/nrc798 12044010

[11] Crosbie EJ, Einstein MH, Franceschi S, Kitchener HC. Human papillomavirus and cervical cancer. Lancet. 2013;382: 889–899. 10.1016/S0140-6736(13)60022-7 23618600

[12] Muñoz N, Bosch FX, De Sanjosé S, Herrero R, Castellsagué X, Shah K V., et al. Epidemiologic classification of human papillomavirus types associated with cervical cancer. N Engl J Med. 2003;348: 518–527. 10.1056/NEJMoa021641 12571259

[13] Culp TD, Budgeon LR, Christensen ND. Human papillomaviruses bind a basal extracellular matrix component secreted by keratinocytes which is distinct from a membrane-associated receptor. Virology. 2006;347: 147–159. 10.1016/j.virol.2005.11.025 16376962

[14] Selinka H-C, Florin L, Patel HD, Freitag K, Schmidtke M, Makarov VA, et al. Inhibition of Transfer to Secondary Receptors by Heparan Sulfate-Binding Drug or Antibody Induces Noninfectious Uptake of Human Papillomavirus. J Virol. 2007;81: 10970–10980. 10.1128/JVI.00998-07 17686860 PMC2045555

[15] Guan J, Bywaters SM, Brendle SA, Ashley RE, Makhov AM, Conway JF, et al. Cryoelectron Microscopy Maps of Human Papillomavirus 16 Reveal L2 Densities and Heparin Binding Site. Structure. 2017;25: 253–263. 10.1016/j.str.2016.12.001 28065506

[16] Bienkowska-Haba M, Patel HD, Sapp M. Target cell cyclophilins facilitate human papillomavirus type 16 infection. PLoS Pathog. 2009;5. 10.1371/journal.ppat.1000524 19629175 PMC2709439

[17] Cerqueira C, Liu Y, Kühling L, Chai W, Hafezi W, van Kuppevelt TH, et al. Heparin increases the infectivity of Human Papillomavirus Type 16 independent of cell surface proteoglycans and induces L1 epitope exposure. Cell Microbiol. 2013;15: 1818–1836. 10.1111/cmi.12150 23601855 PMC4731924

[18] Becker M, Greune L, Schmidt MA, Schelhaas M. Extracellular conformational changes in the capsid of human papillomaviruses contribute to asynchronous uptake into host cells. J Virol. 2018;92: JVI.02106–17. 10.1128/JVI.02106-17 29593032 PMC5952151

[19] Cerqueira C, Samperio Ventayol P, Vogeley C, Schelhaas M. Kallikrein-8 Proteolytically Processes Human Papillomaviruses in the Extracellular Space To Facilitate Entry into Host Cells. J Virol. 2015;89: 7038–7052. 10.1128/JVI.00234-15 25926655 PMC4473586

[20] Bronnimann MP, Calton CM, Chiquette SF, Li S, Lu M, Chapman JA, et al. Furin Cleavage of L2 during Papillomavirus Infection: Minimal Dependence on Cyclophilins. J Virol. 2016;90: 6224–6234. 10.1128/JVI.00038-16 27122588 PMC4936150

[21] Scheffer KD, Gawlitza A, Spoden GA, Zhang XA, Lambert C, Berditchevski F, et al. Tetraspanin CD151 Mediates Papillomavirus Type 16 Endocytosis. J Virol. 2013;87: 3435–3446. 10.1128/JVI.02906-12 23302890 PMC3592167

[22] Spoden G, Freitag K, Husmann M, Boller K, Sapp M, Lambert C, et al. Clathrin- and caveolin-independent entry of human papillomavirus type 16—Involvement of tetraspanin-enriched microdomains (TEMs). PLoS One. 2008;3. 10.1371/journal.pone.0003313 18836553 PMC2561052

[23] Bannach C, Brinkert P, Kühling L, Greune L, Schmidt MA, Schelhaas M. Epidermal growth factor receptor and Abl2 kinase regulate distinct steps of Human papillomavirus type 16 endocytosis. J Virol. 2020; JVI.02143–19. 10.1128/JVI.02143-19 32188731 PMC7269448

[24] Schelhaas M, Shah B, Holzer M, Blattmann P, Kühling L, Day PM, et al. Entry of human papillomavirus type 16 by actin-dependent, clathrin- and lipid raft-independent endocytosis. PLoS Pathog. 2012;8. 10.1371/journal.ppat.1002657 22536154 PMC3334892

[25] Bienkowska-Haba M, Williams C, Kim SM, Garcea RL, Sapp M. Cyclophilins Facilitate Dissociation of the Human Papillomavirus Type 16 Capsid Protein L1 from the L2/DNA Complex following Virus Entry. J Virol. 2012;86: 9875–9887. 10.1128/JVI.00980-12 22761365 PMC3446629

[26] DiGiuseppe S, Bienkowska-Haba M, Hilbig L, Sapp M. The nuclear retention signal of HPV16 L2 protein is essential for incoming viral genome to transverse the trans-Golgi network. Virology. 2014;458–459: 93–105. 10.1016/j.virol.2014.04.024 24928042 PMC4115330

[27] Day PM, Thompson CD, Schowalter RM, Lowy DR, Schiller JT. Identification of a Role for the trans-Golgi Network in Human Papillomavirus 16 Pseudovirus Infection. J Virol. 2013;87: 3862–3870. 10.1128/JVI.03222-12 23345514 PMC3624235

[28] DiGiuseppe S, Bienkowska-Haba M, Guion LGM, Keiffer TR, Sapp M. Human Papillomavirus Major Capsid Protein L1 Remains Associated with the Incoming Viral Genome throughout the Entry Process. Banks L, editor. J Virol. 2017;91: e00537–17. 10.1128/JVI.00537-17 28566382 PMC5533910

[29] Day PM, Weisberg AS, Thompson CD, Hughes MM, Pang YY, Lowy DR, et al. Human Papillomavirus 16 Capsids Mediate Nuclear Entry during Infection. J Virol. 2019;93: 1–18. 10.1128/JVI.00454-19 31092566 PMC6639283

[30] Zhang P, Monteiro da Silva G, Deatherage C, Burd C, DiMaio D. Cell-Penetrating Peptide Mediates Intracellular Membrane Passage of Human Papillomavirus L2 Protein to Trigger Retrograde Trafficking. Cell. 2018;174: 1465–1476.e13. 10.1016/j.cell.2018.07.031 30122350 PMC6128760

[31] Lipovsky A, Popa A, Pimienta G, Wyler M, Bhan A, Kuruvilla L, et al. Genome-wide siRNA screen identifies the retromer as a cellular entry factor for human papillomavirus. Proc Natl Acad Sci U S A. 2013;110: 7452–7457. 10.1073/pnas.1302164110 23569269 PMC3645514

[32] Pim D, Broniarczyk J, Bergant M, Playford MP, Banks L. A Novel PDZ Domain Interaction Mediates the Binding between Human Papillomavirus 16 L2 and Sorting Nexin 27 and Modulates Virion Trafficking. J Virol. 2015;89: 10145–10155. 10.1128/JVI.01499-15 26202251 PMC4580170

[33] Bergant M, Banks L. SNX17 Facilitates Infection with Diverse Papillomavirus Types. J Virol. 2013;87: 1270–1273. 10.1128/JVI.01991-12 23115288 PMC3554065

[34] Zhang W, Kazakov T, Popa A, DiMaio D. Vesicular trafficking of incoming human papillomavirus 16 to the Golgi apparatus and endoplasmic reticulum requires γ-Secretase Activity. MBio. 2014;5: 1–11. 10.1128/mBio.01777-14 25227470 PMC4172078

[35] Inoue T, Zhang P, Zhang W, Goodner-Bingham K, Dupzyk A, DiMaio D, et al. γ-Secretase promotes membrane insertion of the human papillomavirus L2 capsid protein during virus infection. J Cell Biol. 2018;217: 3545–3559. 10.1083/jcb.201804171 30006461 PMC6168257

[36] Popa A, Zhang W, Harrison MS, Goodner K, Kazakov T, Goodwin EC, et al. Direct Binding of Retromer to Human Papillomavirus Type 16 Minor Capsid Protein L2 Mediates Endosome Exit during Viral Infection. PLoS Pathog. 2015;11: 1–21. 10.1371/journal.ppat.1004699 25693203 PMC4334968

[37] Pyeon D, Pearce SM, Lank SM, Ahlquist P, Lambert PF. Establishment of human papillomavirus infection requires cell cycle progression. PLoS Pathog. 2009;5. 10.1371/journal.ppat.1000318 19247434 PMC2642596

[38] Aydin I, Weber S, Snijder B, Samperio Ventayol P, Kühbacher A, Becker M, et al. Large Scale RNAi Reveals the Requirement of Nuclear Envelope Breakdown for Nuclear Import of Human Papillomaviruses. PLoS Pathog. 2014;10. 10.1371/journal.ppat.1004162 24874089 PMC4038628

[39] DiGiuseppe S, Luszczek W, Keiffer TR, Bienkowska-Haba M, Guion LGM, Sapp MJ. Incoming human papillomavirus type 16 genome resides in a vesicular compartment throughout mitosis. Proc Natl Acad Sci U S A. 2016;113: 6289–6294. 10.1073/pnas.1600638113 27190090 PMC4896702

[40] Calton CM, Bronnimann MP, Manson AR, Li S, Chapman JA, Suarez-Berumen M, et al. Translocation of the papillomavirus L2/vDNA complex across the limiting membrane requires the onset of mitosis. PLoS Pathog. 2017;13: 1–29. 10.1371/journal.ppat.1006200 28463988 PMC5412990

[41] Aydin I, Villalonga-Planells R, Greune L, Bronnimann MP, Calton CM, Becker M, et al. A central region in the minor capsid protein of papillomaviruses facilitates viral genome tethering and membrane penetration for mitotic nuclear entry. PLoS Pathogens. 2017. 10.1371/journal.ppat.1006308 28464022 PMC5412989

[42] DiGiuseppe S, Keiffer TR, Bienkowska-Haba M, Luszczek W, Guion LGM, Müller M, et al. Topography of the Human Papillomavirus Minor Capsid Protein L2 during Vesicular Trafficking of Infectious Entry. J Virol. 2015;89: 10442–10452. 10.1128/JVI.01588-15 26246568 PMC4580179

[43] Schneider MA, Spoden GA, Florin L, Lambert C. Identification of the dynein light chains required for human papillomavirus infection. Cell Microbiol. 2011;13: 32–46. 10.1111/j.1462-5822.2010.01515.x 21166973

[44] Florin L, Becker KA, Lambert C, Nowak T, Sapp C, Strand D, et al. Identification of a Dynein Interacting Domain in the Papillomavirus Minor Capsid Protein L2. J Virol. 2006;80: 6691–6696. 10.1128/JVI.00057-06 16775357 PMC1488977

[45] Nagai M, Yoneda Y, Yoneda Y, Yoneda Y. Small GTPase Ran and Ran-binding proteins. Biomol Concepts. 2012;3: 307–318. 10.1515/bmc-2011-0068 25436539

[46] Schulze H, Dose M, Korpal M, Meyer I, Italiano JE, Shivdasani RA. RanBP10 is a cytoplasmic guanine nucleotide exchange factor that modulates noncentrosomal microtubules. J Biol Chem. 2008;283: 14109–14119. 10.1074/jbc.M709397200 18347012 PMC2376235

[47] Kunert S, Meyer I, Fleischhauer S, Wannack M, Fiedler J, Shivdasani RA, et al. The microtubule modulator RanBP10 plays a critical role in regulation of platelet discoid shape and degranulation. Blood. 2009;114: 5532–5540. 10.1182/blood-2009-04-216804 19801445

[48] Meyer I, Kunert S, Schwiebert S, Hagedorn I, Italiano JE, Dütting S, et al. Altered microtubule equilibrium and impaired thrombus stability in mice lacking RanBP10. Blood. 2012;120: 3594–3602. 10.1182/blood-2012-01-401737 22936655

[49] Harada N, Yokoyama T, Yamaji R, Nakano Y, Inui H. RanBP10 acts as a novel coactivator for the androgen receptor. Biochem Biophys Res Commun. 2008;368: 121–125. 10.1016/j.bbrc.2008.01.072 18222118

[50] Day PM, Baker CC, Lowy DR, Schiller JT. Establishment of papillomavirus infection is enhanced by promyelocytic leukemia protein (PML) expression. Proc Natl Acad Sci U S A. 2004;101: 14252–14257. 10.1073/pnas.0404229101 15383670 PMC521143

[51] Day PM, Roden RBS, Lowy DR, Schiller JT. The Papillomavirus Minor Capsid Protein, L2, Induces Localization of the Major Capsid Protein, L1, and the Viral Transcription/Replication Protein, E2, to PML Oncogenic Domains. J Virol. 1998;72: 142–150. 10.1128/JVI.72.1.142-150.1998 9420209 PMC109358

[52] Buck CB, Thompson CD, Pang Y-YS, Lowy DR, Schiller JT. Maturation of Papillomavirus Capsids. J Virol. 2005;79: 2839–2846. 10.1128/JVI.79.5.2839-2846.2005 15709003 PMC548454

[53] Biryukov J, Meyers C. Papillomavirus infectious pathways: A comparison of systems. Viruses. 2015;7: 4303–4325. 10.3390/v7082823 26247955 PMC4576184

[54] Scholey JM, Sharp DJ, Rogers GC. Microtubule motors in mitosis. Nature. 2000;407: 41–47. Available: http://www.nature.com/doifinder/10.1038/35024000%0Apapers3://publication/doi/10.1038/35024000 10993066 10.1038/35024000

[55] Okada N, Sato M. Spatiotemporal Regulation of Nuclear Transport Machinery and Microtubule Organization. Cells. 2015;4: 406–426. 10.3390/cells4030406 26308057 PMC4588043

[56] Schatz CA, Santarella R, Hoenger A, Karsenti E, Mattaj IW, Gruss OJ, et al. Importin α-regulated nucleation of microtubules by TPX2. EMBO J. 2003;22: 2060–2070. 10.1093/emboj/cdg195 12727873 PMC156067

[57] Ciciarello M, Mangiacasale R, Thibier C, Guarguaglini G, Marchetti E, Di Fiore B, et al. Importin β is transported to spindle poles during mitosis and regulates Ran-dependent spindle assembly factors in mammalian cells. J Cell Sci. 2004;117: 6511–6522. 10.1242/jcs.01569 15572412

[58] Giesecke A, Stewart M. Novel binding of the mitotic regulator TPX2 (target protein for Xenopus kinesin-like protein 2) to importin-α. J Biol Chem. 2010;285: 17628–17635. 10.1074/jbc.M110.102343 20335181 PMC2878527

[59] Darshan MS, Lucchi J, Harding E, Moroianu J. The L2 Minor Capsid Protein of Human Papillomavirus Type 16 Interacts with a Network of Nuclear Import Receptors. J Virol. 2004;78: 12179–12188. 10.1128/JVI.78.22.12179-12188.2004 15507604 PMC525100

[60] Carazo-Salas RE, Guarguaglini G, Gruss OJ, Segref A, Karsenti E, Mattaj LW. Generation of GTP-bound ran by RCC1 is required for chromatin-induced mitotic spindle formation. Nature. 1999;400: 178–181. 10.1038/22133 10408446

[61] Ciciarello M, Mangiacasale R, Lavia P. Spatial control of mitosis by the GTPase Ran. Cell Mol Life Sci. 2007;64: 1891–1914. 10.1007/s00018-007-6568-2 17483873 PMC11136129

[62] Chen WS, Chen YJ, Huang YA, Hsieh BY, Chiu HC, Kao PY, et al. Ran-dependent TPX2 activation promotes acentrosomal microtubule nucleation in neurons. Sci Rep. 2017;7: 1–15. 10.1038/s41598-016-0028-x 28205572 PMC5304320

[63] Plafker K, Macara IG. Facilitated Nucleocytoplasmic Shuttling of the Ran Binding Protein RanBP1. Mol Cell Biol. 2000;20: 3510–3521. 10.1128/mcb.20.10.3510-3521.2000 10779340 PMC85643

[64] Delphin C, Guan T, Melchior F, Gerace L. RanGTP targets p97 to RanBP2, a filamentous protein localized at the cytoplasmic periphery of the nuclear pore complex. Mol Biol Cell. 1997;8: 2379–2390. 10.1091/mbc.8.12.2379 9398662 PMC25714

[65] Deane R, Schäfer W, Zimmermann HP, Mueller L, Görlich D, Prehn S, et al. Ran-binding protein 5 (RanBP5) is related to the nuclear transport factor importin-beta but interacts differently with RanBP1. Mol Cell Biol. 1997;17: 5087–5096. 10.1128/mcb.17.9.5087 9271386 PMC232359

[66] Döhner K, Wolfstein A, Prank U, Echeverri C, Dujardin D, Vallee R, et al. Function of dynein and dynactin in herpes simplex virus capsid transport. Mol Biol Cell. 2002;13: 2795–2809. 10.1091/mbc.01-07-0348 12181347 PMC117943

[67] Bouchard P, Penningroth SM, Cheung A, Gagnon C, Bardin CW. Erythro-9-[3-(2-hydroxynonyl)]adenine is an inhibitor of sperm motility that blocks dynein ATPase and protein carboxylmethylase activities. Proc Natl Acad Sci U S A. 1981;78: 1033–1036. 10.1073/pnas.78.2.1033 6453342 PMC319940

[68] Firestone AJ, Weinger JS, Maldonado M, Barlan K, Langston LD, O’Donnell M, et al. Small-molecule inhibitors of the AAA+ ATPase motor cytoplasmic dynein. Nature. 2012;484: 125–129. 10.1038/nature10936 22425997 PMC3321072

[69] Wu H, Maciejewski MW, Takebe S, King SM. Solution structure of the Tctex1 dimer reveals a mechanism for dynein-cargo interactions. Structure. 2005;13: 213–223. 10.1016/j.str.2004.11.013 15698565

[70] Lo KWH, Kogoy JM, Pfister KK. The DYNLT3 light chain directly links cytoplasmic dynein to a spindle checkpoint protein, Bub3. J Biol Chem. 2007;282: 11205–11212. 10.1074/jbc.M611279200 17289665

[71] Bodor A, Radnai L, Hetényi C, Rapali P, Láng A, Kövér KE, et al. DYNLL2 dynein light chain binds to an extended linear motif of myosin 5a tail that has structural plasticity. Biochemistry. 2014;53: 7107–7122. 10.1021/bi500574z 25312846

[72] Mabit H, Nakano MY, Prank U, Saam B, Döhner K, Sodeik B, et al. Intact Microtubules Support Adenovirus and Herpes Simplex Virus Infections. J Virol. 2002;76: 9962–9971. 10.1128/jvi.76.19.9962-9971.2002 12208972 PMC136514

[73] Jacob Y, Badrane H, Ceccaldi P-E, Tordo N. Cytoplasmic Dynein LC8 Interacts with Lyssavirus Phosphoprotein. J Virol. 2000;74: 10217–10222. 10.1128/jvi.74.21.10217-10222.2000 11024152 PMC102062

[74] Chang CC, Chen CJ, Grauffel C, Pien YC, Lim C, Tsai SY, et al. Ran pathway-independent regulation of mitotic Golgi disassembly by Importin-α. Nat Commun. 2019;10: 1–16. 10.1038/s41467-018-07882-8 31541088 PMC6754406

[75] Franker MAM, Hoogenraad CC. Microtubule-based transport -basic mechanisms, traffic rules and role in neurological pathogenesis. J Cell Sci. 2013;126: 2319–2329. 10.1242/jcs.115030 23729742

[76] Vale RD. The molecular motor toolbox for intracellular transport. Cell. 2003;112: 467–480. 10.1016/s0092-8674(03)00111-9 12600311

[77] Mitchison T, Evans L, Schulze E, Kirschner M. Sites of microtubule assembly and disassembly in the mitotic spindle. Cell. 1986;45: 515–527. 10.1016/0092-8674(86)90283-7 3708686

[78] Meunier S, Vernos I. Microtubule assembly during mitosis—from distinct origins to distinct functions? J Cell Sci. 2012;125: 2805–2814. 10.1242/jcs.092429 22736044

[79] Janisch KM, Vock VM, Fleming MS, Shrestha A, Grimsley-Myers CM, Rasoul BA, et al. The vertebrate-specific Kinesin-6, Kif20b, is required for normal cytokinesis of polarized cortical stem cells and cerebral cortex size. Development. 2013/10/30. 2013;140: 4672–4682. 10.1242/dev.093286 24173802 PMC3833427

[80] Abaza A, Soleilhac J-M, Westendorf J, Piel M, Crevel I, Roux A, et al. M phase phosphoprotein 1 is a human plus-end-directed kinesin-related protein required for cytokinesis. J Biol Chem. 2003/05/11. 2003;278: 27844–27852. 10.1074/jbc.M304522200 12740395 PMC2652640

[81] Miki H, Okada Y, Hirokawa N. Analysis of the kinesin superfamily: Insights into structure and function. Trends Cell Biol. 2005;15: 467–476. 10.1016/j.tcb.2005.07.006 16084724

[82] Schneider MA, Scheffer KD, Bund T, Boukhallouk F, Lambert C, Cotarelo C, et al. The Transcription Factors TBX2 and TBX3 Interact with Human Papillomavirus 16 (HPV16) L2 and Repress the Long Control Region of HPVs. J Virol. 2013;87: 4461–4474. 10.1128/JVI.01803-12 23388722 PMC3624339

[83] Bund T, Spoden GA, Koynov K, Hellmann N, Boukhallouk F, Arnold P, et al. An L2 SUMO interacting motif is important for PML localization and infection of human papillomavirus type 16. Cell Microbiol. 2014;16: 1179–1200. 10.1111/cmi.12271 24444361

[84] Sato Y, Kato A, Maruzuru Y, Oyama M, Kozuka-Hata H, Arii J, et al. Cellular Transcriptional Coactivator RanBP10 and Herpes Simplex Virus 1 ICP0 Interact and Synergistically Promote Viral Gene Expression and Replication. J Virol. 2016;90: 3173–3186. 10.1128/JVI.03043-15 26739050 PMC4810668

[85] Her LS, Mao SH, Chang CY, Cheng PH, Chang YF, Yang HI, et al. miR-196a enhances neuronal morphology through suppressing RANBP10 to provide neuroprotection in Huntington’s disease. Theranostics. 2017;7: 2452–2462. 10.7150/thno.18813 28744327 PMC5525749

[86] Gustafsson N, Culley S, Ashdown G, Owen DM, Pereira PM, Henriques R. Fast live-cell conventional fluorophore nanoscopy with ImageJ through super-resolution radial fluctuations. Nat Commun. 2016;7: 1–9. 10.1038/ncomms12471 27514992 PMC4990649

[87] Sage D, Donati L, Soulez F, Fortun D, Schmit G, Seitz A, et al. DeconvolutionLab2: An open-source software for deconvolution microscopy. Methods. 2017;115: 28–41. 10.1016/j.ymeth.2016.12.015 28057586

[88] Bolte S, Cordelières FP. A guided tour into subcellular colocalization analysis in light microscopy. J Microsc. 2006;224: 213–232. 10.1111/j.1365-2818.2006.01706.x 17210054

[89] Cox J, Mann M. MaxQuant enables high peptide identification rates, individualized p.p.b.-range mass accuracies and proteome-wide protein quantification. Nat Biotechnol. 2008;26: 1367–1372. 10.1038/nbt.1511 19029910

