# Supplementary figures and images for: A Ran-binding protein facilitates nuclear import of human papillomavirus type 16
Source: PLoS Pathog. 2021 May 11;17(5):e1009580. doi: 10.1371/journal.ppat.1009580 (PMC8139508; doi:10.1371/journal.ppat.1009580)

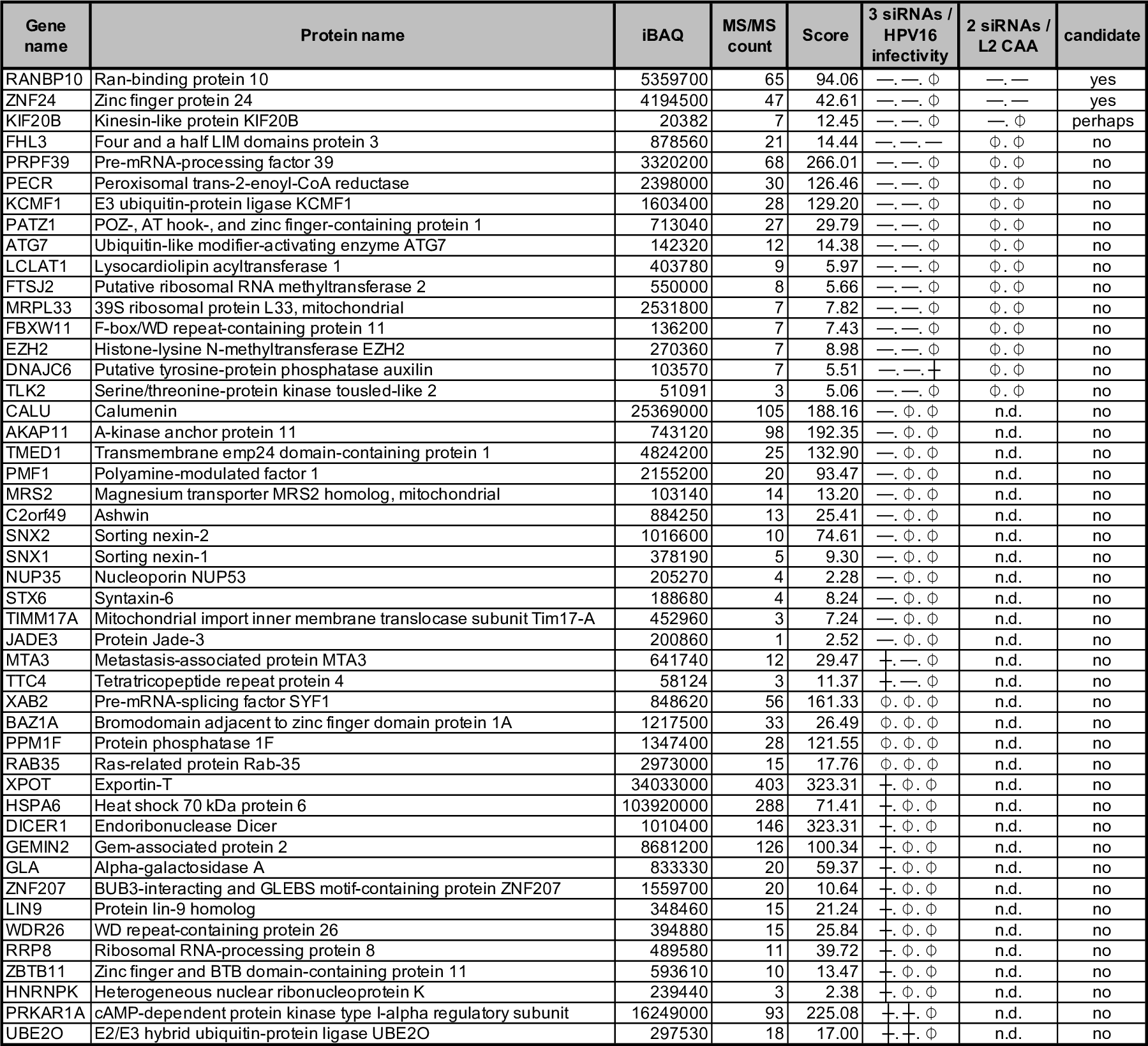

Supplement: S1 Table — The label-free semi-quantitative mass spectrometry and statistical analysis were performed as described in material and methods. Columns: iBAQ (intensity based absolute quantification) represents the total intensities divided by the number of theoretical peptides, i.e. a value proportional to the molar quantities of a protein, thereby providing a rough estimate of the abundance of a protein in each sample. MS/MS count references the total sequenced events for a peptide. Score refers to the Andromeda score for the best associated MS/MS spectrum. The 47 potential cellular interactors of L2 were individually depleted in HeLa cells with three independent siRNA followed by HPV16 infectivity assays or L2 CAA with two siRNAs in HeLa Kyoto_H2B-mCherry_L2-GFP cells, with (—) at least 50% reduction, (┼) at least 50% increase, ⏀: no significant phenotype, n.d.: not determined. Candidates exhibited 50% reduction in both assays for at least two siRNAs. (TIFF) [file ppat.1009580.s002.tiff]

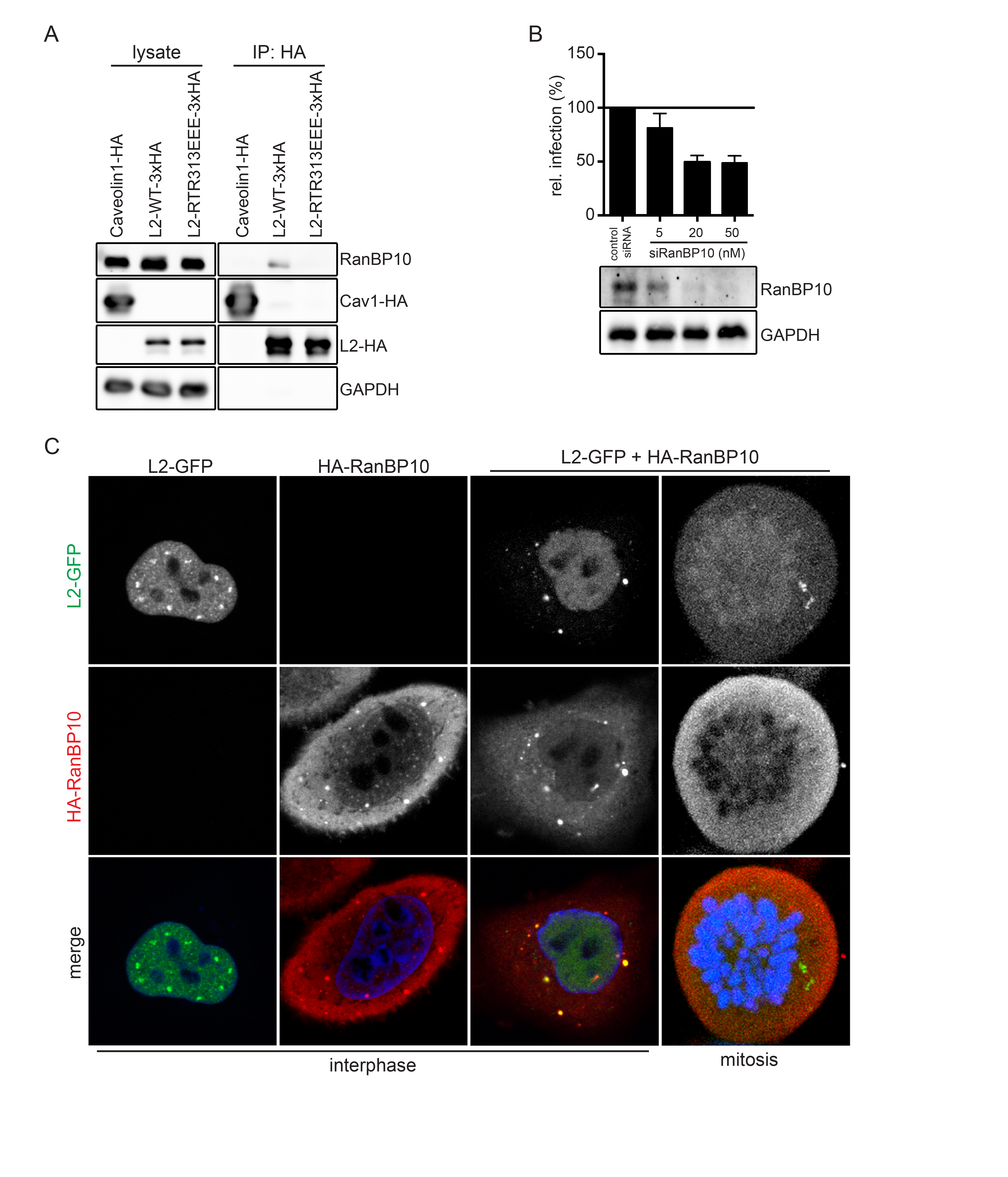

Supplement: S1 Fig — (A) Caveolin1-HA, L2-WT-3x-HA, and L2-RTR313EEE-3xHA were individually expressed in mitotic HEK293 cells to perform immunoprecipitation assay. Caveolin1-HA was used as a negative control. Endogenous RanBP10 and HA-tag were detected by western blotting. (B) RNAi of RanBP10 in HeLa cells using different amounts of siRNA was followed by HPV16-PsV infection for 48 hours. Infectivity was scored by flow cytometry based on the percentage of the cells expressing GFP. The infectivity was normalized to control siRNA transfected cells and depicted as relative (rel.) infection. The protein expression level of RanP10 upon siRNA knockdown was analyzed by Western Blotting. (C) HeLa cells were co-transfected with HA-RanBP10 and/or L2-GFP expressing plasmids. Nucleus was stained with Hoechst-33258. Images were acquired with LSM800 in 700 nm single slices. Images were presented in single median slices. (TIF) [file ppat.1009580.s003.tif]

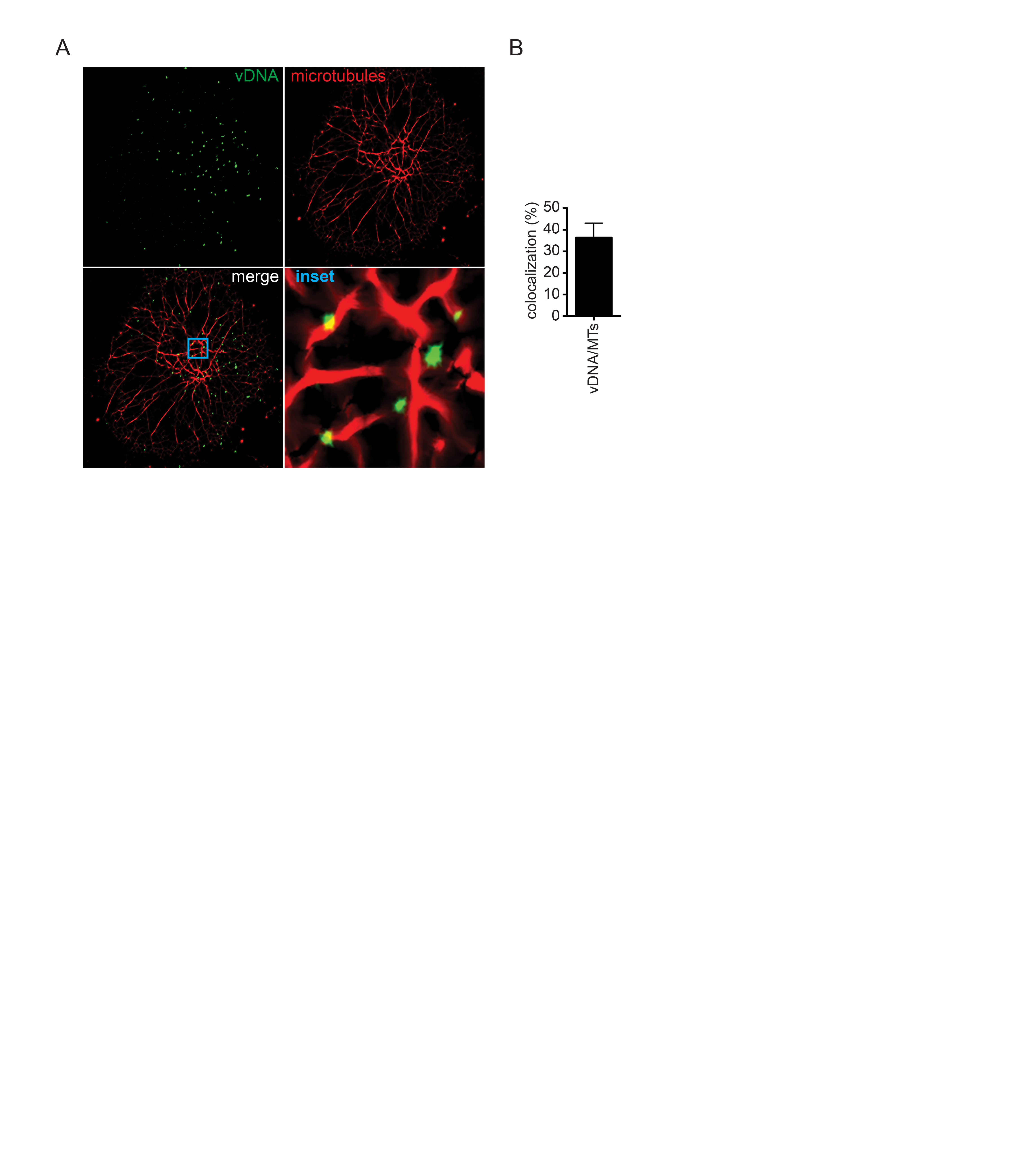

Supplement: S2 Fig — (A) Co-localization of incoming vDNA and MTs. HeLa cells were infected with EdU-labelled HPV16 and arrested in mitosis. Cells were stained for vDNA and alpha-tubulin (MTs). Cells were analyzed by SRRF microscopy as described in material and methods. (B) Quantification of co-localized vDNA with MTs in the super-resolved images. Data represents the average of three independent experiments ±SD. (TIF) [file ppat.1009580.s004.tif]

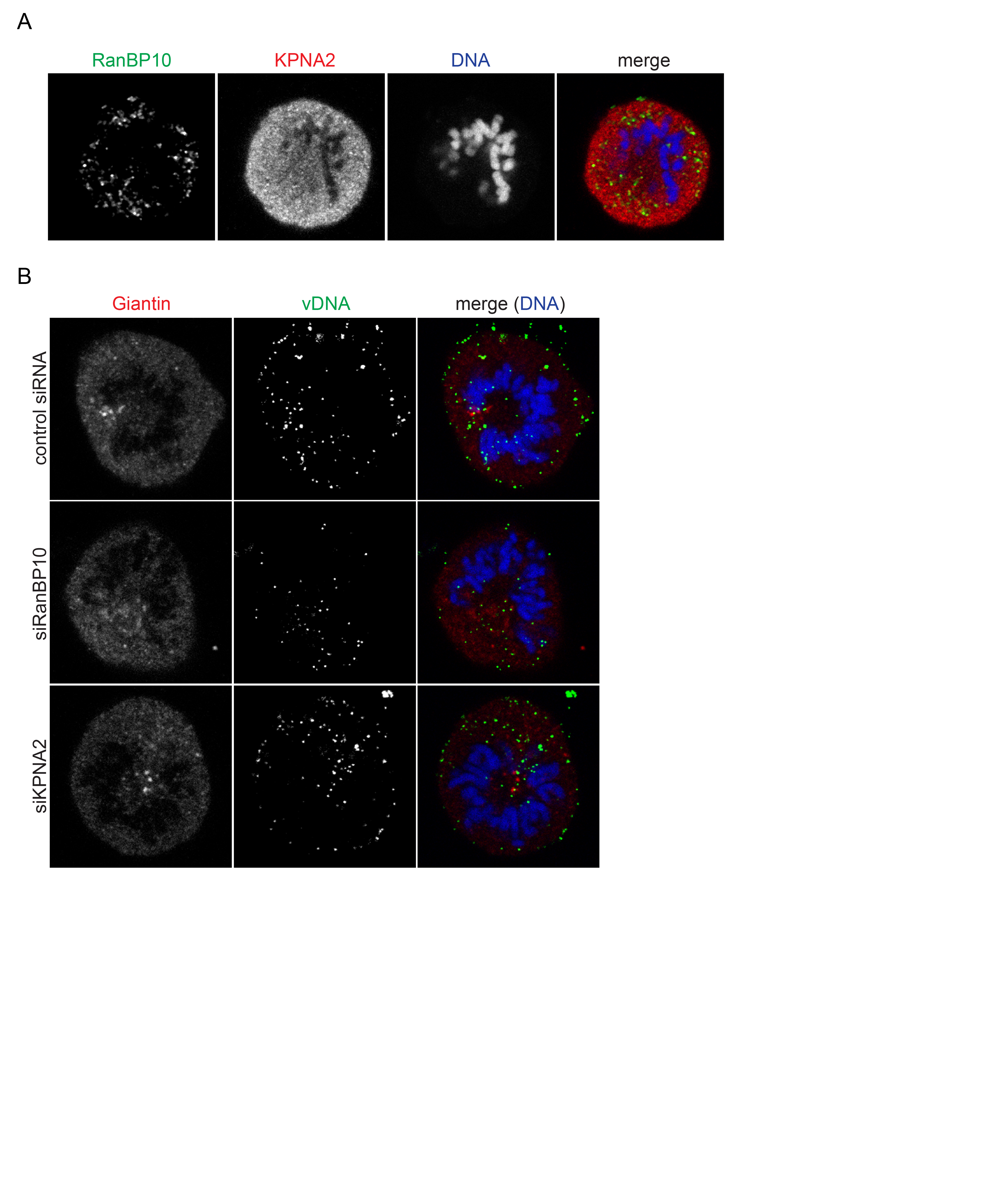

Supplement: S3 Fig — (A) HeLa cells during mitosis stained for endogenous RanBP10, KPNA2, and DNA. (B) HeLa cells with RanBP10 or KPNA2 depletion via RNAi were infected with EdU-labelled HPV16. After 20 h.p.i, cells synchronized in mitosis were fixed and stained with anti-Giantin antibody and Hoechst-33258 to visualize Golgi and mitotic chromosomes. The incoming vDNA labelled with EdU was detected by EdU Click-iT chemistry. Images were acquired by confocal microscopy. Images represent single median slices. (TIF) [file ppat.1009580.s005.tif]

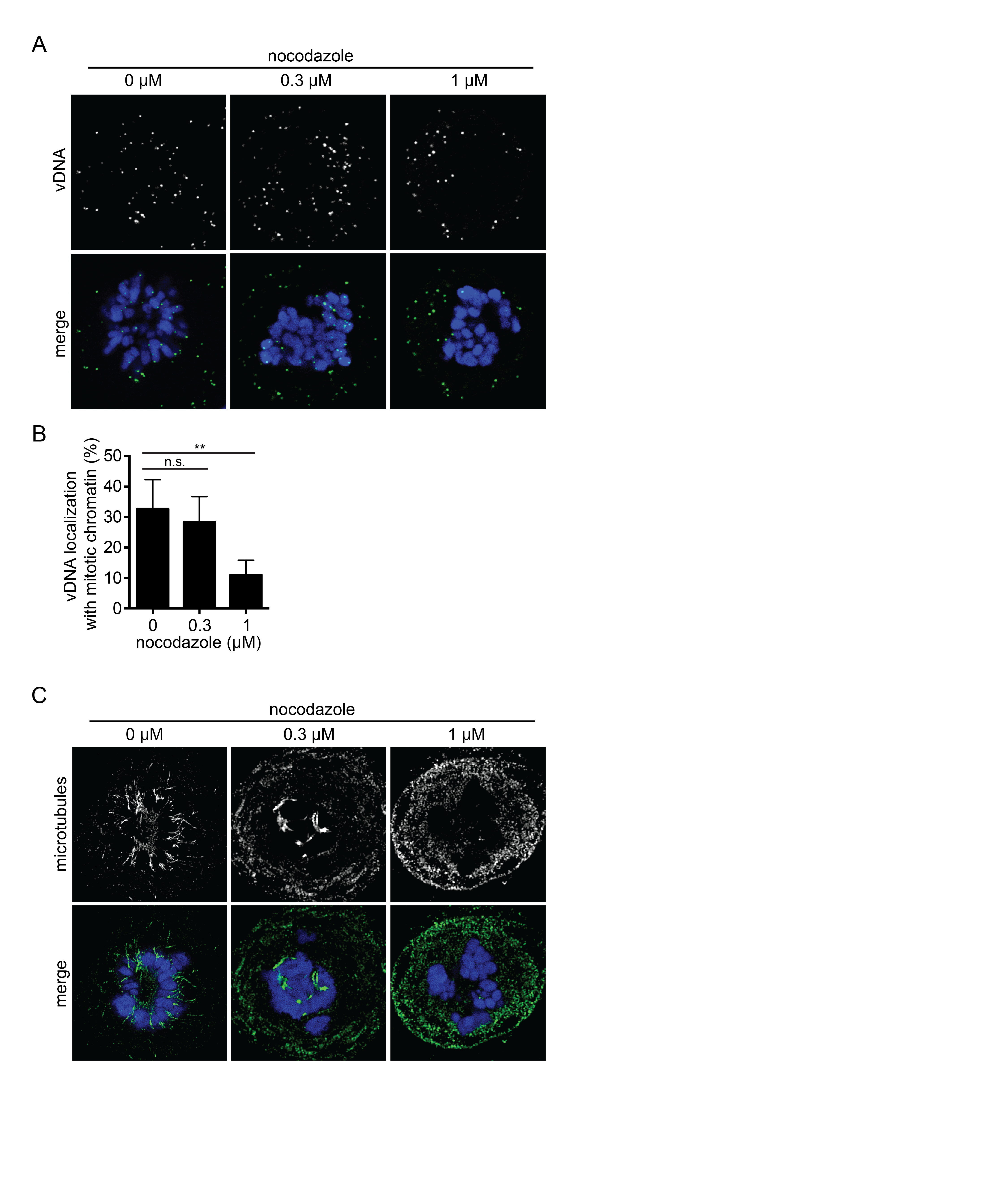

Supplement: S4 Fig — Nocodazole at the different indicated concentrations was used to interfere with MT polymerization. (A) HeLa Kyoto cells were infected with EdU-labelled HPV16 treated with nocodazole at the indicated concentrations two hours prior to mitosis in synchronized cells. Mitotic cells were fixed and vDNA was stained with EdU-Click-iT chemistry. Host DNA was stained with Hoechst-33258 to indicate mitotic chromosomes. Depicted are single confocal slices. (B) Quantification of co-localized vDNA signals with mitotic chromatin upon nocodazole treatments. At least 35 cells were analyzed in three independent experiments. The error bars indicate the SD. n.s.: not significant. (C) HeLa Kyoto cells were treated with nocodazole at the indicated concentrations two hours prior to mitosis in synchronized cells. Mitotic cells were fixed and MTs were stained with an alpha-tubulin antibody. Host DNA was stained with Hoechst-33258 to indicate mitotic chromosomes. Depicted are single confocal slices. (TIF) [file ppat.1009580.s006.tif]

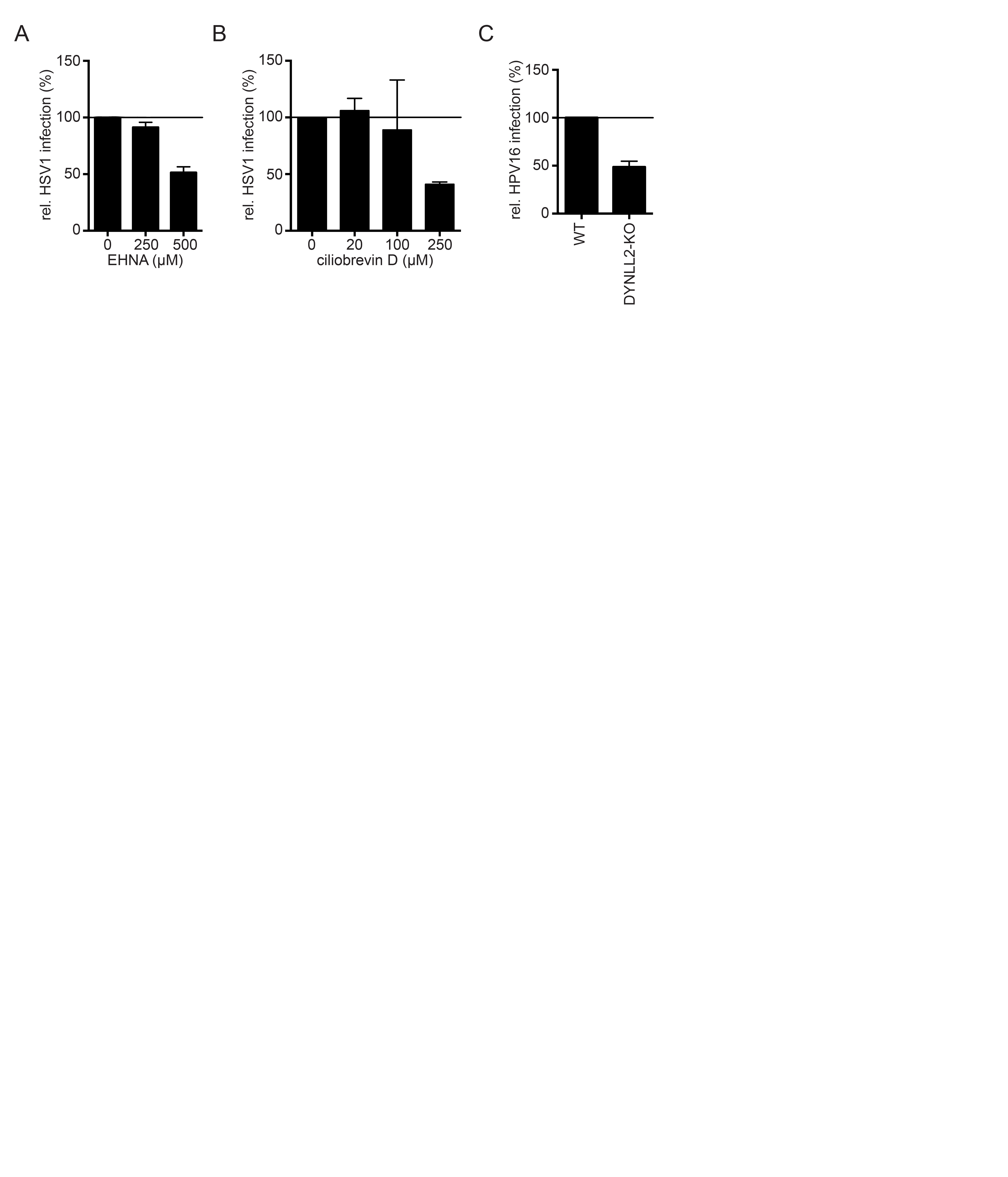

Supplement: S5 Fig — EHNA or ciliobrevin D was used to inhibit dynein-mediated transport. HeLa Kyoto cells were treated with EHNA (A) or ciliobrevin D (B) one hour prior to and during HSV-1-GFP infection. The infectivity was scored based on the percentage of the cells expressing GFP with flow cytometry. The infectivity was normalized to DMSO-treated cells as relative infection. (C) Infection of WT and DYNLL2 CRISPR/Cas9 knockout (KO) HeLa cells. The infectivity was scored based on the percentage of the cells expressing GFP with flow cytometry. The infectivity was normalized to DMSO treated cells as relative infection. (TIF) [file ppat.1009580.s007.tif]

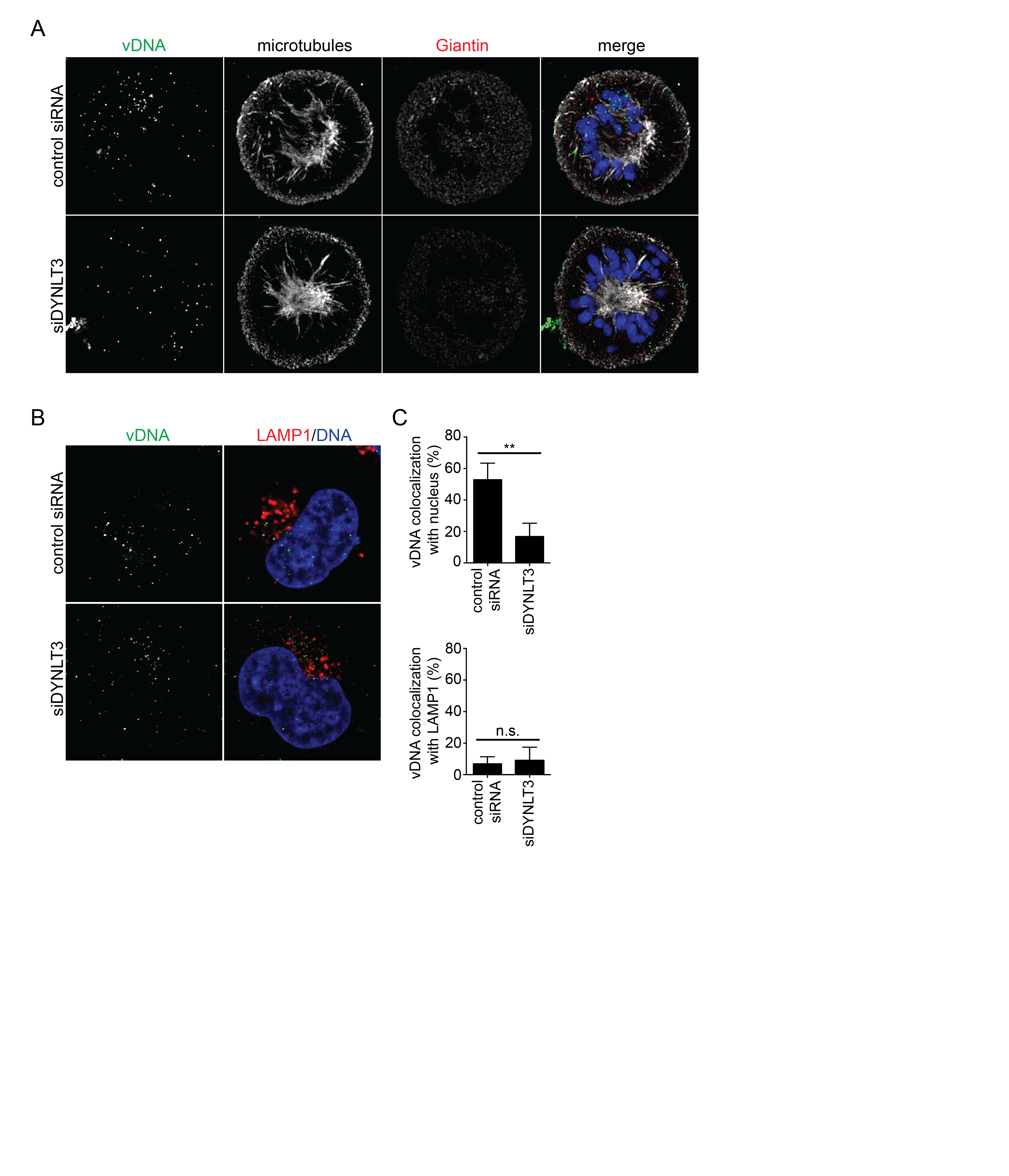

Supplement: S6 Fig — (A) HeLa Kyoto cells treated with siRNA against DYNLT3 or control were infected with EdU-labelled HPV16 and arrested in pro-metaphase as in Fig 2B. Mitotic cells were fixed and vDNA was stained with EdU-Click chemistry. Host DNA was stained with Hoechst-33258 to indicate mitotic chromosomes. Depicted are single confocal slices. (B) HeLa Kyoto cells treated with siRNA against DYNLT3 or control were infected with EdU-labelled HPV16 for 20h. Cells were fixed and vDNA was stained with EdU Click-iT chemistry, and endosomes with an antibody against LAMP1. Host DNA was stained with Hoechst-33258 to indicate cell nuclei. Depicted are single confocal slices. (C) Quantification of co-localized vDNA with cell nuclei or LAMP1 using IMARIS. More than 35 cells were analyzed in three independent experiments, error bars indicate the SD. (TIF) [file ppat.1009580.s008.tif]

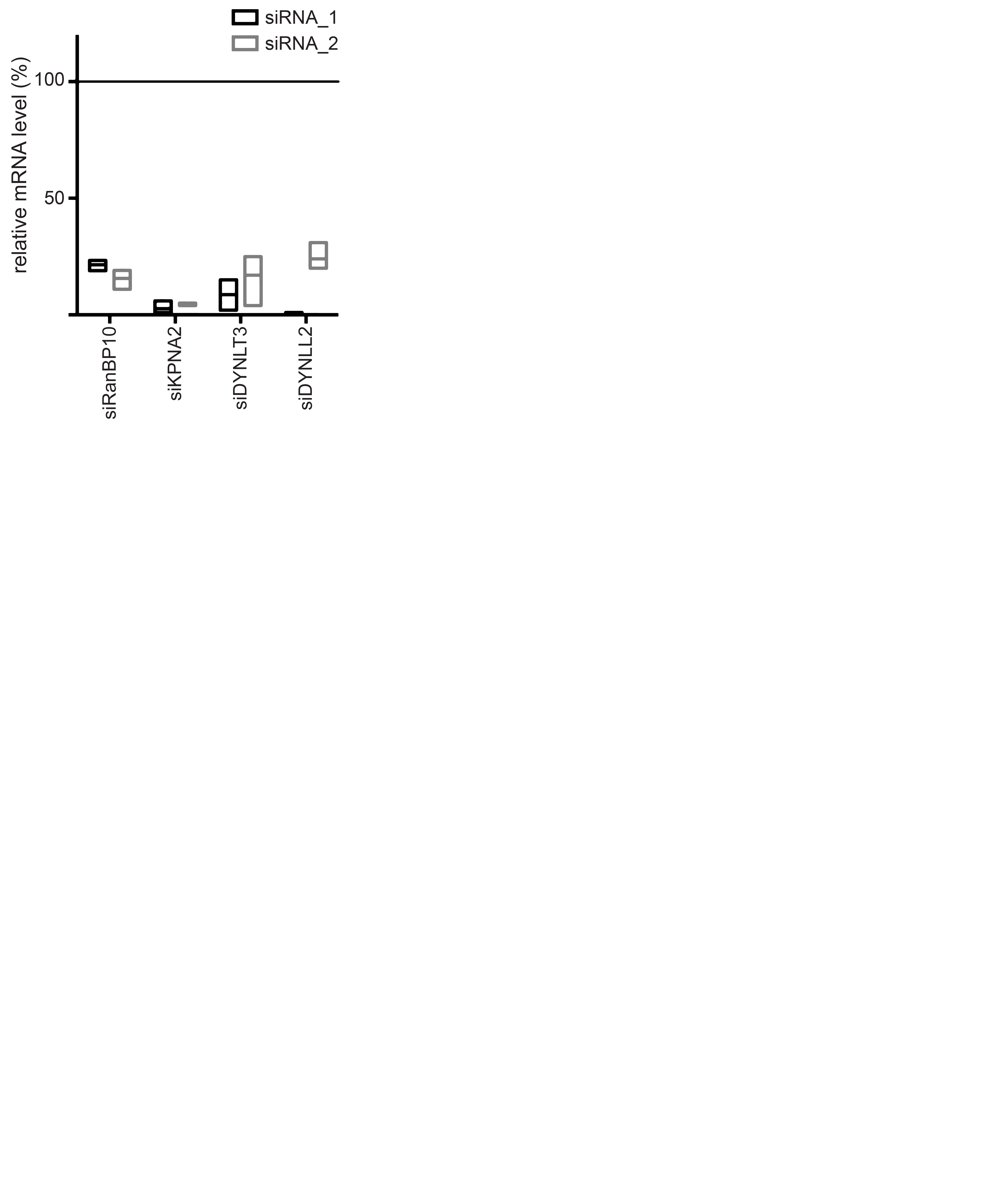

Supplement: S7 Fig — RNAi was performed on HeLa cells as described in materials and methods. The total RNA was extracted with RNA extraction kit from Qiagen (RNeasy kit), and reverse transcription was performed with poly-T primer. Real-time PCR was used to detect the mRNA level of RNAi target protein, as well as GAPDH for normalization. All samples were normalized to control siRNA transfected cells to obtain relative mRNA expression levels. RNAi experiments were performed individually three times. (TIF) [file ppat.1009580.s009.tif]
